# Enhancing detection of low‑abundance metabolites in proton NMR through band‑selective suppression and presaturation

**DOI:** 10.1007/s13659-025-00570-3

**Published:** 2026-01-11

**Authors:** Upendra Singh, Renad Z. Al Ahmadi, Ruba Al‑Nemi, Manel Dhahri, Mohammed S. Alarawi, Abdul Aziz, Faisal Abdulaziz Bushulaybi, Tamer Abdalla Mashtoly, Abdul‑Hamid Emwas, Lukasz Jaremko, Mariusz Jaremko

**Affiliations:** 1 Department of Biochemistry & Molecular Biology (BMB), Sealy Institute for Drug Discovery (SIDD), University of Texas Medical Branch (UTMB), Galveston, TX 77555‑1068 USA; 2 Division of Biological and Environmental Sciences and Engineering (BESE), King Abdullah University of Science and Technology (KAUST), 23955‑6900 Thuwal, Makkah Saudi Arabia; 3 Department of Biology, College of Science, Taibah University, Yanbu Governorate, Saudi Arabia; 4 Computational Bioscience Research Center, KAUST, 23955‑6900 Thuwal, Makkah Saudi Arabia; 5 National Center for Palms and Dates, Prince Turki Ibn Abdulaziz Al Awwal Rd, Hittin, 13512 Riyadh, Saudi Arabia; 6 WEQAA Center, Al-Rabwa, 12813 Riyadh, Saudi Arabia; 7 Plant Protection Department Faculty of Agriculture, Ain Shams University, Hadayek Shoubra, P.O 11241, Cairo, Egypt; 8 Core Lab of NMR, KAUST, 23955‑6900 Thuwal, Makkah Saudi Arabia; 9 The Golden Ratio Institute, 13244 Riyadh, Saudi Arabia

**Keywords:** NMR spectroscopy, Signal-enhancement, Band-selective inversion, Sugars, Metabolomics

## Abstract

Metabolomics provides powerful means to analyze metabolite profiles in biological samples, enabling insights into biochemical changes under genetic, environmental, or pathological conditions. Nuclear Magnetic Resonance (NMR) spectroscopy is central to metabolomics, but its utility is often constrained by the strong and overlapping resonances of abundant components, such as sugars in plant‑ and food‑derived materials, which obscure signals of lower‑abundance metabolites. Here, we introduce a modified NMR acquisition method that increases sensitivity and specificity by selectively suppressing dominant signals, while enhancing weaker metabolite signals across the spectrum. The method integrates water presaturation with excitation sculpting (ES), yielding a robust 1D presat‑^1^H‑ES pulse sequence. Validation on a range of sugar-rich samples demonstrated 2–fourfold signal enhancement for low‑abundance metabolites compared with conventional ^1^H‑ES. Multivariate analyses show the method improves reproducibility and discrimination, enabling detection and comparison of low‑abundance metabolites not accessible with conventional approaches’. Beyond sugar‑rich systems, the method is broadly applicable to other spectral regions where dominant metabolite classes obscure lower‑concentration compounds, including primary metabolites and structurally diverse natural products. Overall, the 1D presat‑^1^H‑ES significantly enhances resolution and sensitivity of NMR‑based metabolomics, shortens analysis time, and supports more precise profiling for both fundamental studies and translational applications in metabolomics and natural‑products discovery.

**Graphical Abstract:**

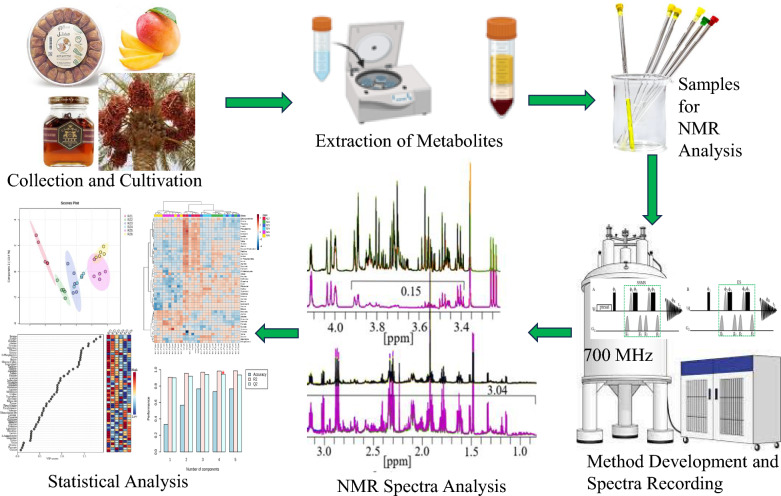

**Supplementary Information:**

The online version contains supplementary material available at 10.1007/s13659-025-00570-3.

## Highlights

- Robust 1D presat‑^1^H‑ES NMR method integrating presaturation and excitation sculpting to selectively suppress dominant signals.
- Validated on plant‑derived and food materials, improving sensitivity, reproducibility, and multivariate discrimination.
- Fast, user‑friendly, high‑throughput solution for metabolic profiling with broad applications in food quality, nutrition, and natural‑product research.

**Supplementary Information:**

The online version contains supplementary material available at 10.1007/s13659-025-00570-3.

## Introduction

Metabolomics offers a systematic framework to profile small‑molecule metabolites in biological systems, yielding insights into physiology, pathology, biomarker discovery, early disease detection, drug discovery, and therapeutic monitoring [1–12]. In plants and food matrices, metabolomics has been used to catalogue species metabolomes, monitor stress‑ and infection‑induced biochemical responses, distinguish genotypes, and map chemical diversity and bioactivity [2, 13–19]. Nuclear Magnetic Resonance (NMR) spectroscopy is a valuable platform for these studies because it is highly reproducible, non‑destructive, and well suited to complex mixtures [20–27], yet it has intrinsically lower sensitivity than mass spectrometry [23, 28]. This limitation is exacerbated in sugar‑rich samples (e.g., dates, honey, fruit juices, and many plant extracts), where intense, overlapping ^1^H NMR sugar resonances dominate spectra, masking low‑abundance primary and secondary metabolites (natural products) and reducing accuracy of quantitation and multivariate analyses [29, 30].

Over the past decade, convergent advances in hardware, hyperpolarization, micro‑detection, and have substantially increased NMR sensitivity and selectivity for metabolite analysis in complex biological matrices [31–33]. Key strategies include detuned cryoprobes [34], suppression of selected regions [29, 35], dynamic nuclear polarization [36, 37], microcoils coupled with photochemically induced dynamic nuclear polarization (DNP) [38], ultra‑fast (UF) methods [39], and optimized solvent‑suppression and filtering sequences such as WATERGATE [40], CPMG filtering [41], and one‑dimensional band‑selective excitation sculpting (1D ^1^H‑ES) [42]. The Suppression of Sugar's Moiety Signals (SSMS) approach [43] was developed to attenuate dominant sugar resonances and enhance detection of low‑abundance metabolites, but it requires extended measurement time and calibration, limiting throughput. To address this, we developed a hybrid pulse sequence: the presat‑^1^H‑ES that integrates water presaturation with band‑selective excitation sculpting to suppress selected dominant resonances. Proposed presat‑^1^H‑ES delivers 2–4 times enhancement of target‑metabolite sensitivity, achieves stronger sugar signals suppression while preserving adjacent chemical shifts, and reduces residual water signal to negligible levels without increasing total experiment time and baseline artefacts.

We validate presat-^1^H-ES in quantitative metabolomic profiling of selected plant-based and food products, including date flesh at three distinct ripening stages: Khalal (unripe), Rutab (half-ripe), and Tamr (fully ripe), as well as in mango pulp and honey samples. Comparative analyses demonstrate superior accuracy, precision, reproducibility, and multivariate discrimination relative to conventional 1D ^1^H-ES, enabling reliable detection of low-abundance biomarkers in high-sugar matrices and significantly reducing acquisition time and associated costs.

Here, presat-^1^H-ES represents a robust, high-throughput solution for NMR-based metabolomics of sugar-rich samples, establishing a new benchmark for rapid, cost-effective analysis of complex sugar rich biological mixtures.

## Materials and methods

### Sample preparations

#### Date flesh sample

Dates were sourced from a supermarket in Saudi Arabia. A 100-mg portion of date flesh was ground in a stone pestle and mortar in liquid nitrogen, dissolved in 1 mL of 80% methanol-in-water solution, and homogenized using a thermomixer comfort (Eppendorf 1.5 mL) at 1,400 rpm for 45 min at 4 °C. The resulting suspension was centrifuged at 15,000 × *g* for 10 min at 4 °C. Subsequently, 800 µL of the supernatant was lyophilized in a vacuum concentrator (CentriVap, Labconco, Kansas City, MO, USA) for 8 h at 35 °C to yield pellets. The lyophilized pellets were then reconstituted in 600 µL of 20 mM deuterium oxide solution containing potassium phosphate buffer (pH 7.4) and 0.5 mM TSP as an internal standard reference. Each suspended solution was vortexed for complete dissolution and centrifuged at 15,000 × *g* for 10 min at 4 °C, and a 550-µL aliquot of the clear solution was transferred to a 5-mm NMR tube for NMR analysis.

#### Mango flesh sample

Mangoes were sourced from a supermarket in Saudi Arabia. The inner slices were finely ground in a stone pestle and mortar in liquid nitrogen. The moisture from the resulting powder was removed via lyophilization using a vacuum pressure machine (Labconco, 4.5 L, − 105 °C). A 70-mg portion of the dried powder was dissolved in 1 mL of 80% methanol-in-water solution and homogenized on a thermomixer comfort (Eppendorf 1.5 mL) at 14,00 rpm for 45 min at 4 °C. The resulting suspension was centrifuged at 15,000 × *g* for 10 min at 4 °C, after which 800 µL of the supernatant was lyophilized in a vacuum concentrator for 8 h at 35 °C to yield pellets. These pellets were reconstituted in 600 µL of 20 mM deuterium oxide solution containing potassium phosphate buffer (pH 7.4) and 0.5 mM TSP. The suspended solutions were vortexed for complete dissolution and centrifuged at 15,000 × *g* for 10 min at 4 °C. Then, 550 µL of each clear solution was transferred to a 5-mm NMR tube for NMR analysis.

#### Honey sample

Honey products were procured from a supermarket in Saudi Arabia. For each sample, 100 mg was individually dissolved in 600 µL of deuterium oxide containing 0.5 mM TSP. The suspended solutions were vortexed for 5 min and centrifuged at 15,000 × *g* for 10 min at 4 °C, after which 550 µL of each clarified solution was transferred to a 5-mm NMR tube for subsequent NMR analysis.

#### Cultivated date flesh sample

Ajwa date was cultivated at three different stages (Khalal [half-ripe], Rutab [soft-ripened], and Tamr [fully ripened] to validate the modified NMR method and for metabolic profiling of these different stages. Samples were prepared similarly to date flesh.

### NMR spectroscopy

A Bruker 700 MHz AVANACE NEO NMR spectrometer equipped with a Bruker TXO (^1^H/^13^C/^15^N/^2^H) cryogenic probe (Bruker, Billerica, MA, USA) was used to record all NMR spectra at 298 K. The transformed spectra were processed for phase and baseline distortions using Topspin 4.1.4 (Bruker) and then calibrated to the proton signal of TSP at 0.00 ppm. The same acquisition parameters were used for both 1D ^1^H-ES and presat-^1^H-ES NMR. For each spectrum, free induction decays (FIDs) were collected at 55,554 data points from 128 scans with four dummy scans at a spectral width of 13,888.889 Hz, an acquisition time of 2 s, and a relaxation delay of 5 s. Both 1D ^1^H-ES and presat-^1^H-ES NMR used the same experimental time of approximately 15.5 min. The processing parameters were 131072 data points for zero filling, and an exponential window function was applied to the FID with a line-broadening factor of 0.3 Hz and SSB of 2 before Fourier transformation. Parameters were adjusted according to the sample preparations to permit the quantification of metabolite annotations using the Chenomx NMR Suite 9.0 profiler (Chenomx, Edmonton, Canada).

### Pulse sequences of 1D presat-^1^H-ES and 1D ^1^H-ES

**Fig. 1:**
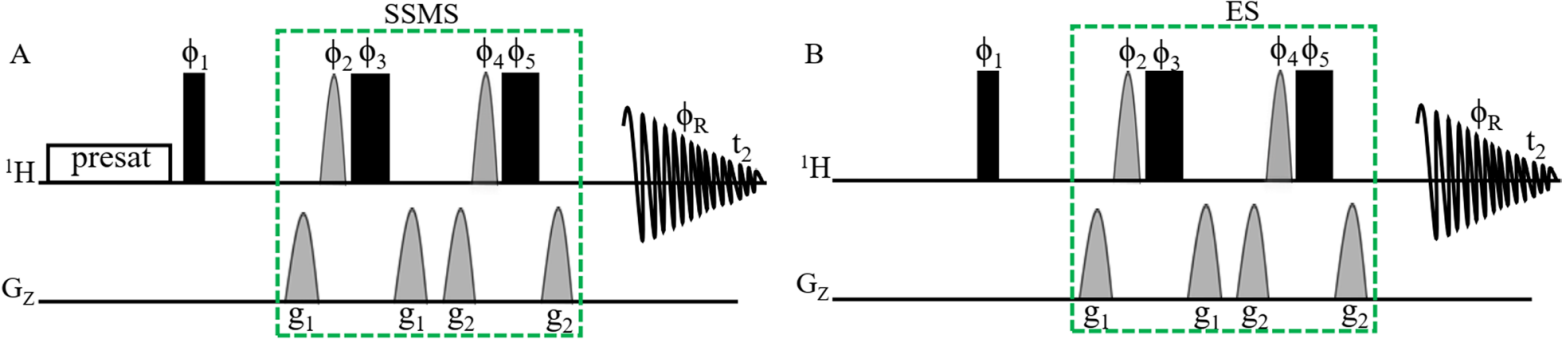
**A** Pulse sequence of the 1D presat-^1^H-ES method. In this pulse sequence, water presaturation was combined with conventional ^1^H-ES. Narrow and filled rectangular black bars represent hard 90° and 180° pulses, respectively. Light, gray half-sine shapes represent soft 180° pulses (Sinc 1.1000 for 2 ms). These pulses have phases such as f_1_ = x; f_2_ = x, y; f_3_ =  − x, − y; f_4_ = x, x, y, y; f_5_ =  − x, − x, − y, − y; and f_R_ = x, − x, − x, x. g_1_ = 31% for 1 ms and g_2_ = 11% for 1 ms are pulse-field gradients used to remove artifacts. **B** The pulse sequence of the 1D ^1^H-ES method

Figure 1 presents the pulse sequences of the modified 1D presat-^1^H-ES hybrid method (Fig. 1A) and the original 1D ^1^H-ES method (Fig. 1B). In the new method, water presaturation is combined with ^1^H-ES, and the application of ES is shifted from water suppression to SSMS, for which sugar signals in the 3.20–4.00 ppm region was considered dominant sugar signals. Band-selective shape pulse Sinc1.1000 is commonly used to achieve the selective inversion of the magnetization of water molecules in ^1^H-ES, but in the new hybrid method, the band-selective shape pulse was used to achieve the selective inversion of the magnetization of dominant sugar signals.

The ES block was an efficient arrangement of pulse sequences for water suppression in studies of different types of complex mixtures in deuterated solvents. When the ES block is applied, water magnetizations remain unperturbed, whereas the rest of the magnetizations are perturbed. Therefore, this block is applied to suppress dominant sugar signals following the same concept of the remaining unperturbed magnetizations of dominant signals and the perturbed magnetizations of low‑abundance signals. Presaturation using a continuous wave of pulse sequences for water suppression is also a commonly used technique in the 1D ^1^H NMR method to achieve water suppression using the presat-^1^H-ES NMR method.

## Results and discussion

### Comparison of 1D presat-^1^H-ES NMR method with 1D ^1^H-ES in date flesh

**Fig. 2:**
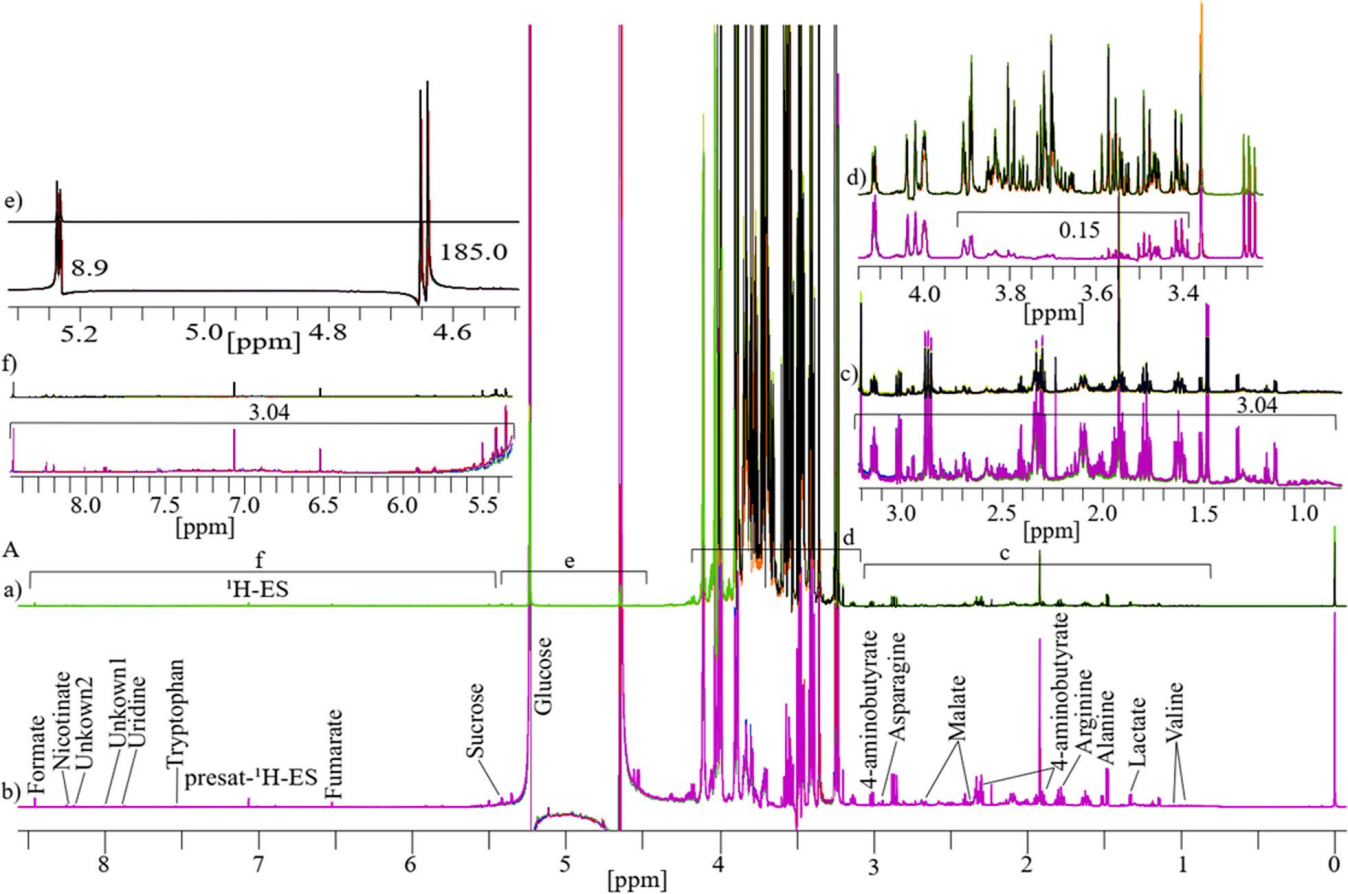
**A** The typical 1D ^1^H NMR spectra consisting of four replicates obtained from date flesh samples and labeled as **a** for ^1^H-ES and **b** for presat-^1^H-ES. The date flesh samples were extracted in a polar solution and dissolved in deuterium oxide containing phosphate buffer. Each full spectrum was divided into four sections, which covered chemical shift regions spanning 0.80–3.20 (**c**), 3.25–4.15 (**d**), 4.50–5.32 (**e**), and 5.40–8.49 ppm (**f**). Nineteen signals corresponding to identified and unidentified metabolites were specifically chosen across the entire spectrum for quantification purposes. The analysis of these signals was based on spectra acquired using both methods to facilitate comparisons between them

The results for four replicates of date flesh analyzed through 1D ^1^H-ES and presat-^1^H-ES NMR are presented in Fig. 2A. The ^1^H-ES spectrum exhibited complexities attributable to the high concentration of sugars and relatively low concentrations of amino acids, organic compounds, and DNA biomolecules (bases and nucleotides). In presat-^1^H-ES, a novel method aiming to suppress sugar signals while enhancing the peak intensities of desired molecules (Fig. 2b), nineteen peaks of identified and unidentified metabolites were selectively assigned across the entire spectra of the 1D ^1^H-ES and presat-^1^H-ES methods to compare the suppression capacity and enhancements of all signal intensities. Estimation of the factors of suppression and enhancement is presented in the divided subsections of Fig. 2A in the expanded regions of spectra from both NMR methods, namely, 0.85–3.20 ppm in Fig. 2c, 3.22–4.15 ppm in Fig. 2d, 4.50–5.34 ppm in Fig. 2e, and 5.35–8.48 ppm in Fig. 2f. Through its suppression approach, presat-^1^H-ES was found to enhance the peak intensities in the spectrum within the range of 0.85–3.20 ppm by 3.04-fold compared with ^1^H-ES (Fig. 2c). The factor of all signal intensities of the ^1^H-ES spectrum is considered as a unit. The residual signal from suppressed sugars within the range of 3.40–3.90 ppm was reduced to 0.15-fold in the spectrum of presat-^1^H-ES relative to the unit intensities of peaks of the ^1^H-ES spectrum (Fig. 2d). Notably, the peak intensities of β- and α-glucose at 4.60 and 5.20 ppm increased by 185.0- and 8.9-fold, respectively (Fig. 2e). In addition, a 3.04-fold increase in peak intensities was observed within the 5.39–8.45-ppm region (Fig. 2f).

**Fig. 3:**
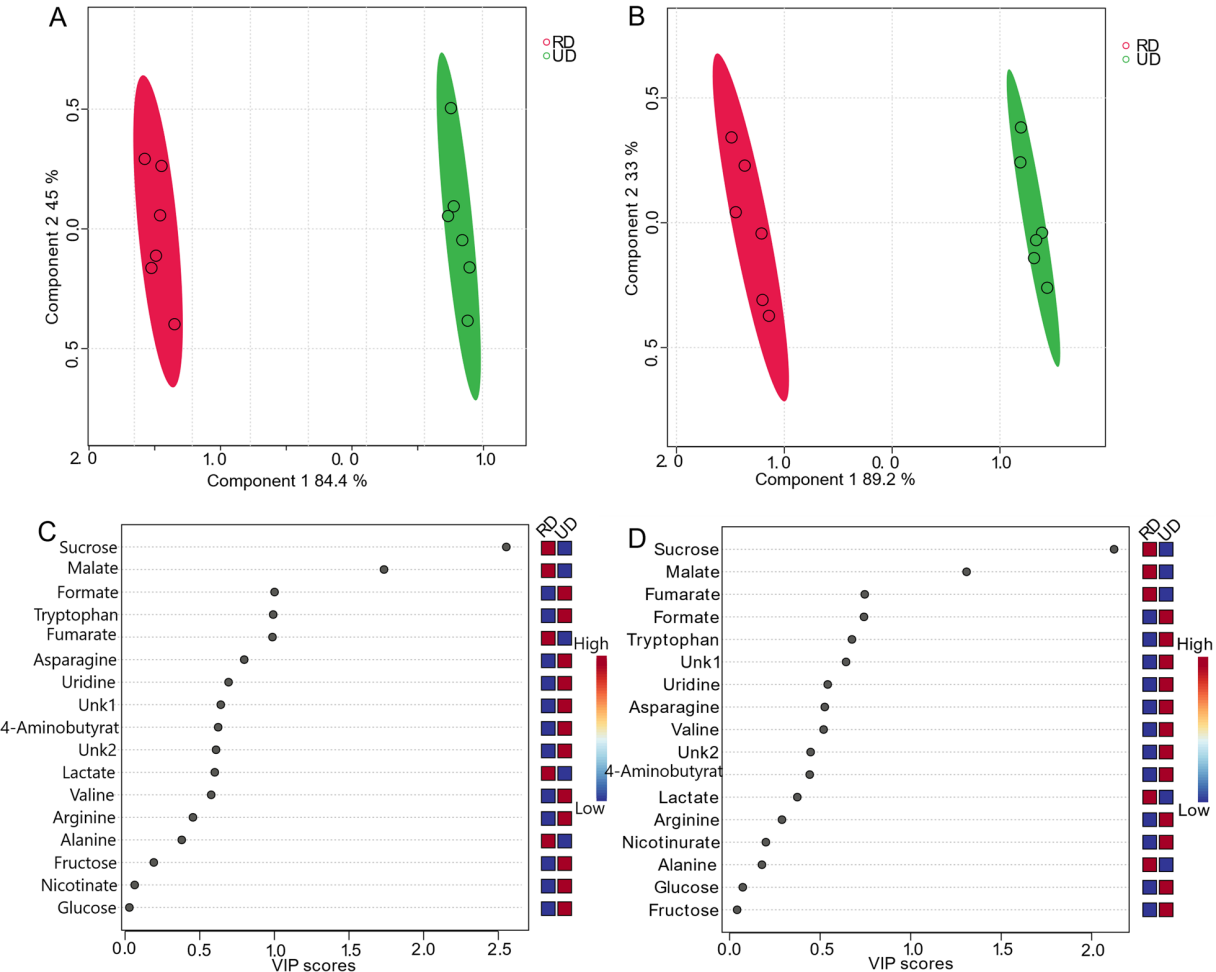
**a** PLS-DA based on metabolomics data from two types of date flesh samples (RD [red, n = 6] and UD [green, n = 6]) with six replicates (n) obtained using 1D ^1^H-ES NMR. **b** PLS-DA score plot based on the same samples but using 1D presat-^1^H-ES NMR. VIP score charts, calculated as the weighted sum of squares of the PLS-DA loadings considering the amount of explained Y-variation in each dimension based on data acquired from **c** 1D ^1^H-ES and **d** presat-^1^H-ES NMR spectra

For multivariate analysis, six replicates were used for both the presat-^1^H-ES and ^1^H-ES 1D ^1^H NMR spectra. Various multivariate methods, such as score plots, variable importance in projection (VIP) analysis, heatmaps, and cross-validation, were employed through partial least squares-discriminate analysis (PLS-DA) for both NMR methods. The 2D score plots generated from PLS-DA using data from 1D ^1^H-ES and presat-^1^H-ES demonstrated significant clustering of replicates in each group and significant separation between the two varieties of date flesh samples (denoted as RD and UD, respectively; Fig. 3a, b, respectively). Comparing the visual score plots of both NMR methods revealed similar separation trends, but stronger clustering of the groups was observed in presat-^1^H-ES than in ^1^H-ES. In particular, the scores of data from both methods revealed a concentration of score points in components one (89.2% for presat-^1^H-ES vs. 84.4% for ^1^H-ES) and two (3.3% for presat-^1^H-ES vs. 4.5% for ^1^H-ES). PLS-DA represents a robust method for identifying differentially expressed metabolites, primarily utilizing the VIP score. Variables with VIP scores exceeding one are considered crucial in constructing the model. The VIP score plots, generated from two components of PLS-DA, were employed to highlight metabolites that differentiated the RD and UD groups. In the ^1^H-ES data (Fig. 3c), sucrose and malate emerged as the most significant metabolites (VIP scores of 1.7 and 2.5, respectively), exhibiting higher levels in RD than in UD. Similarly, the same VIP scores were recorded for these molecules in the presat-^1^H-ES data (Fig. 3d). Furthermore, the VIP scores were closely aligned between the two NMR methods for the remaining metabolites (Fig. 3c, d). Heatmaps were generated to visualize the metabolomic profiles of the RD and UD groups using data acquired from the NMR spectra of twelve replicates. Figure S1A presents the heatmap constructed from data obtained via 1D ^1^H-ES, whereas Figure S1B presents the heatmap derived from data obtained via 1D presat-^1^H-ES. In the analysis, the ratio of specific metabolites was computed by comparing their concentrations under a given condition to the average concentration across all samples at each time point. Upon comparison, the results revealed similarities between the heatmaps generated by both NMR methods. However, in the heatmap generated from presat-^1^H-ES data, there was a more noticeable homogeneous clustering among replicates within each group compared with the heatmap generated from ^1^H-ES data. This enhanced clustering was achieved by the increased peak intensities, which facilitated more precise annotation in various metabolic processing software.

Cross-validation plays a critical role in ensuring the robustness of models, particularly in methods such as PLS-DA, in which there is a risk of overfitting, given its classification nature [44]. We applied leave-one-out cross-validation (LOOCV) to data from both ^1^H-ES (Figure S2A) and presat-^1^H-ES (Figure S2B), and Q2 was evaluated on five components, resulting in the following values: Q2 = 0.99474, R2 = 0.99988, and accuracy = 1.0 for ^1^H-ES (Figure S2C) and Q2 = 0.99575, R2 = 0.99988, and accuracy = 1.00 for ^1^H-ES data (Figure S2D). In supervised classification models, R2 and Q2 serve as accuracy parameters, with values ranging from 0 to 1; higher values indicate greater accuracy. R2 denotes the raw predictive accuracy of the model. Q2 is derived from constructing a PLS model using a training set and testing it against a separate test set. Typically, a Q2 value exceeding 0.65 is considered significant for a model’s predictability. The data revealed the comparable robustness of both methods when comparing R2, Q2, and accuracy between their respective datasets. Notably, Q2 was negligibly higher for presat-^1^H-ES than for ^1^H-ES.

### Comparison of 1D presat-^1^H-ES NMR method with 1D ^1^H-ES in mango flesh

**Fig. 4:**
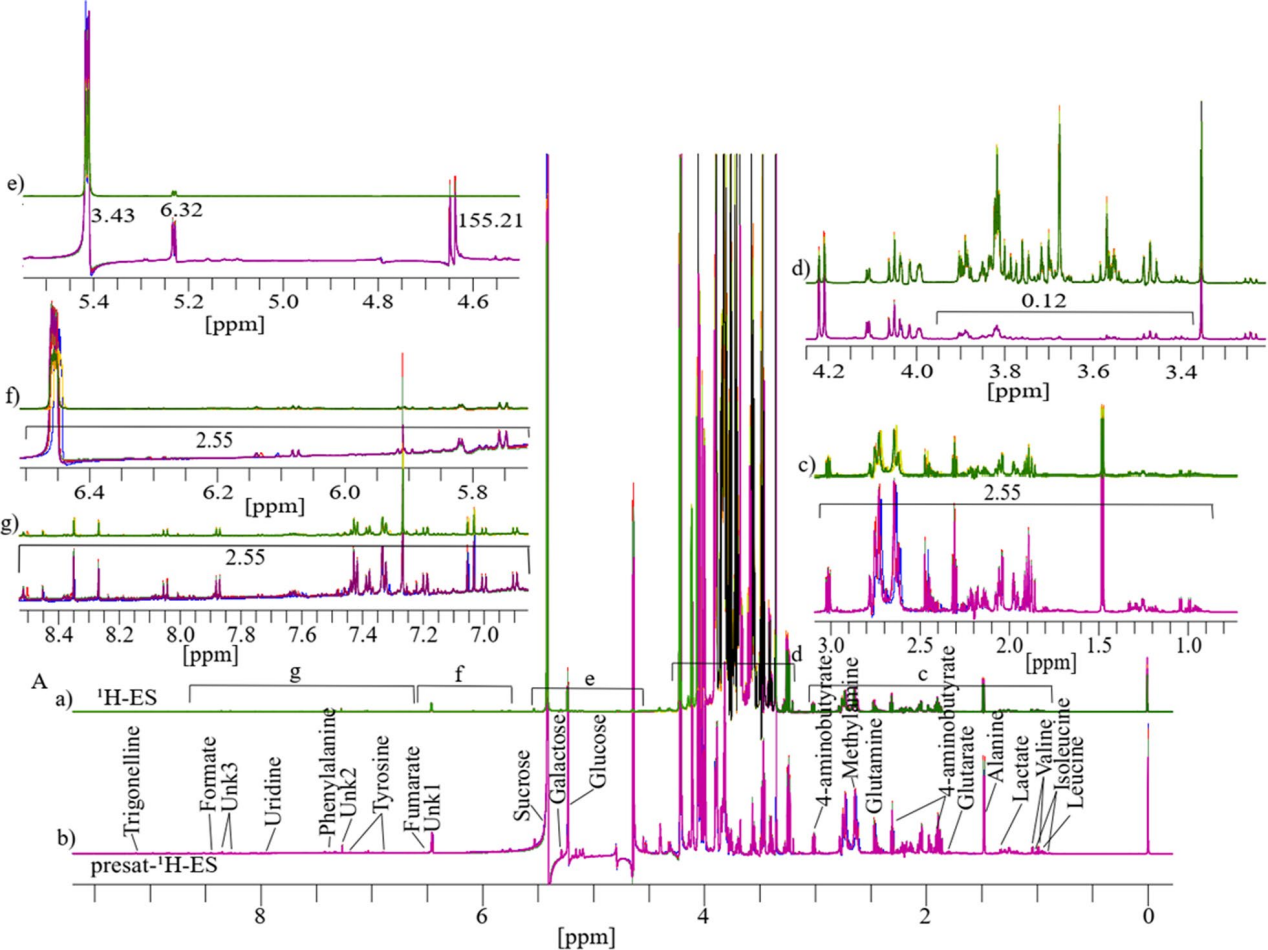
**A** Typical 1D ^1^H NMR full spectra with four replicates, labeled as **a** for ^1^H-ES and **b** for ^1^H-presat-ES. These spectra were obtained from the metabolites of ripe mango flesh samples, which were extracted in a polar solution and dissolved in deuterium oxide containing phosphate buffer. These full spectra were divided into five sections covering the chemical shift regions of 0.80–3.00 (**c**), 3.25–4.25 (**d**), 4.52–5.44 (**e**), 5.72–6.51 (**f**), and 6.87–8.52 ppm (**g**). Twenty-nine peaks corresponding to identified and unidentified metabolites were specifically chosen across the entire spectrum for quantification purposes. These signals were analyzed from spectra acquired using both methods to facilitate comparisons between them

**Fig. 5:**
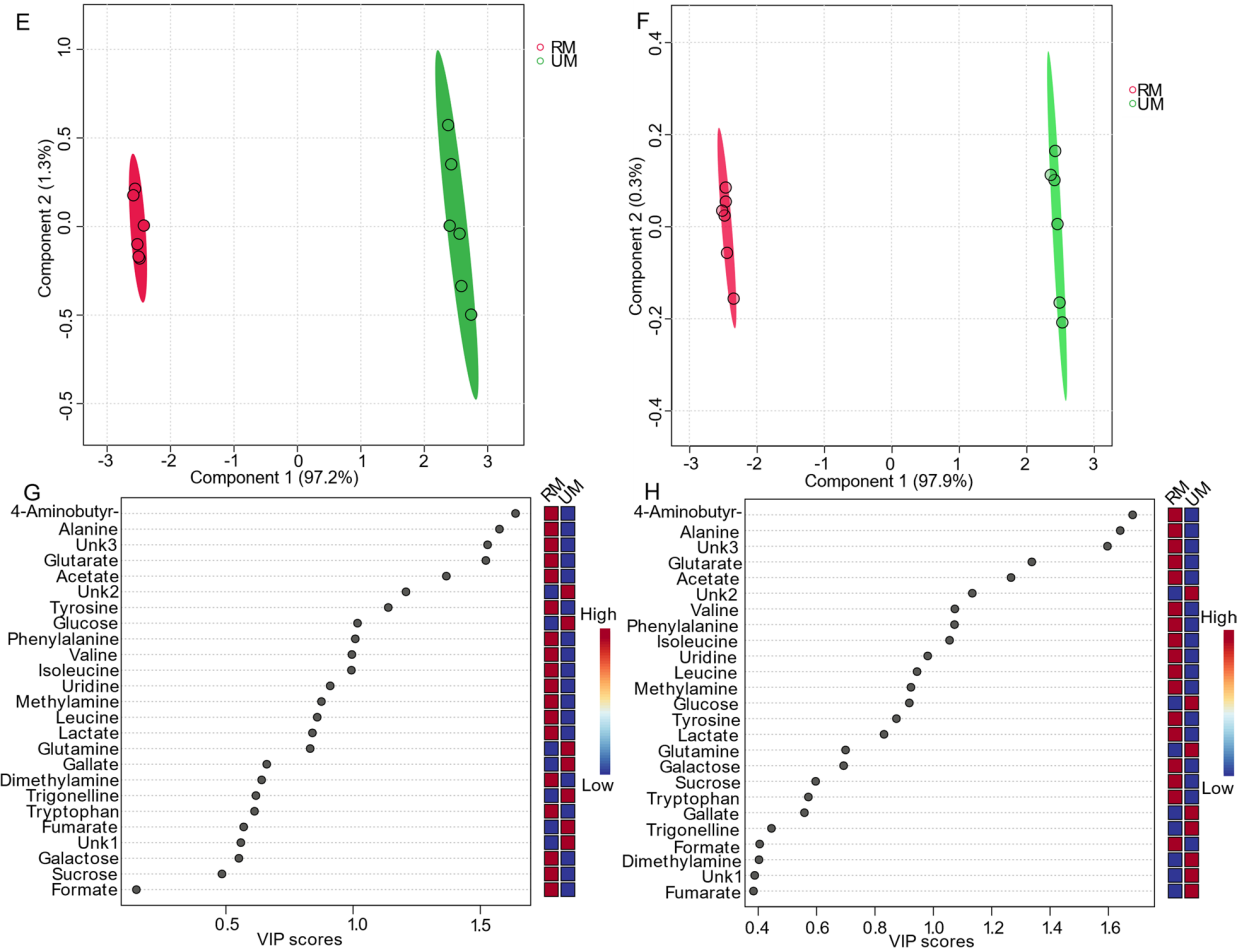
PLS-DA based on metabolomics data from the 1D ^1^H-ES NMR (**e**) and presat-^1^H-ES (**f**) NMR spectra of RM (red, n = 6) and UM (green, n = 6) ripe mango flesh samples with a total of twelve replicates. VIP score charts based on data acquired from 1D ^1^H-ES (**g**) and presat-^1^H-ES (**h**) NMR spectra

Similarly, six replicates of 1D ^1^H-ES and presat-^1^H-ES NMR spectra were obtained using mango flesh (Fig. 4A). The ^1^H-ES spectrum is presented in Fig. 4a, and the presat-^1^H-ES spectrum is presented in Fig. 4b. The new method was employed to suppress the sugar signals while enhancing the peak intensities of desired molecules, resulting in the selective assignment of twenty-nine metabolites across the whole spectrum of presat-^1^H-ES compared with the findings for the conventional ^1^H-ES method. The peak intensities in the expanded spectrum of the presat-^1^H-ES region of 0.75–3.05 ppm were estimated to be 2.55-fold stronger than those of ^1^H-ES (Fig. 4c). In addition, the residual signal from suppressed sugar in the region of 3.40–3.90 ppm was reduced to 0.12-fold of its original intensity in the ^1^H-ES spectrum (Fig. 4d). Notably, the peak intensities of β-glucose, α-glucose, and sucrose were increased by 155.0-, 6.32-, and 3.43-fold, respectively, at chemical shifts of 4.60, 5.20, and 5.40 ppm, respectively (Fig. 4e). Moreover, the peak intensities within the region of 5.75–8.51 ppm were increased by 2.55-fold (Fig. 4f, g). 1D ^1^H NMR spectra with twelve replicates for both presat-^1^H-ES and ^1^H-ES were used for multivariate analysis using score plots, VIP, heatmaps, and cross-validation of PLS-DA data. The 2D score plots of PLS-DA revealed significant separation with considerable clustering among the same replicates between the two groups of ripe mango flesh samples analyzed by processing 1D ^1^H-ES and presat-^1^H-ES data, as presented in Fig. 5a, b, respectively. A comparison of the visual score plots of both NMR methods disclosed almost similar separation and clustering of the groups, whereas slightly higher scores were observed in components one (97.2% vs. 97.9%) and two (1.3% vs. 0.3%) for presat-^1^H-ES. The VIP score plots, generated from the two-component PLS-DA model, were utilized to identify discriminant metabolites between the RM and UM groups. These plots highlighted significant disparities in the concentrations of 4-aminobutyrate, alanine, Unk3 (an unidentified metabolite), glutarate, acetate, Unk2, valine, phenylalanine, isoleucine, uridine, and leucine, with VIP scores consistently surpassing the threshold of one in both ^1^H-ES and presat-^1^H-ES NMR analyses (Fig. 5g, h). Notably, other metabolites exhibited VIP scores exceeding or around one in ^1^H-ES, and presat-^1^H-ES showed similar scores. Eleven key metabolites were found to substantially contribute to both the statistical variance in the metabolomic data and the metabolic divergence observed between the RM and UM mango sample groups (Fig. 5g, h). The remaining metabolites demonstrated comparable VIP scores across both groups, underscoring the analogous reproducibility and analytical robustness of presat-^1^H-ES and ^1^H-ES methodologies. Interestingly, although the overall patterns were comparable, there was a reversal in the clustering within each group when comparing the heatmaps generated using ^1^H-ES (Figure S3A) and presat-^1^H-ES data (Figure S3B). Specifically, the clustering of replicates within the same group was more homogeneous for presat-^1^H-ES than for ^1^H-ES. This discrepancy is attributable to the enhanced peak intensities achieved using the presat-^1^H-ES method, which facilitates more accurate annotation of peaks of metabolites, particularly when employing various types of metabolic processing software. We applied LOOCV to both ^1^H-ES (Figure S4A) and presat-^1^H-ES data (Figure S4B). Q2 was evaluated on five components, resulting in the following values: Q2 = 0.99914, R2 = 0.99982, and accuracy = 1.0 for ^1^H-ES (Figure S4C) and Q2 = 0.99923, R2 = 0.99952, and accuracy = 1.00 for presat-^1^H-ES data (Figure S4D). These results highlighted the similar robustness of both NMR methods.

### Comparison of 1D presat-^1^H-ES NMR method with 1D ^1^H-ES in honey

**Fig. 6:**
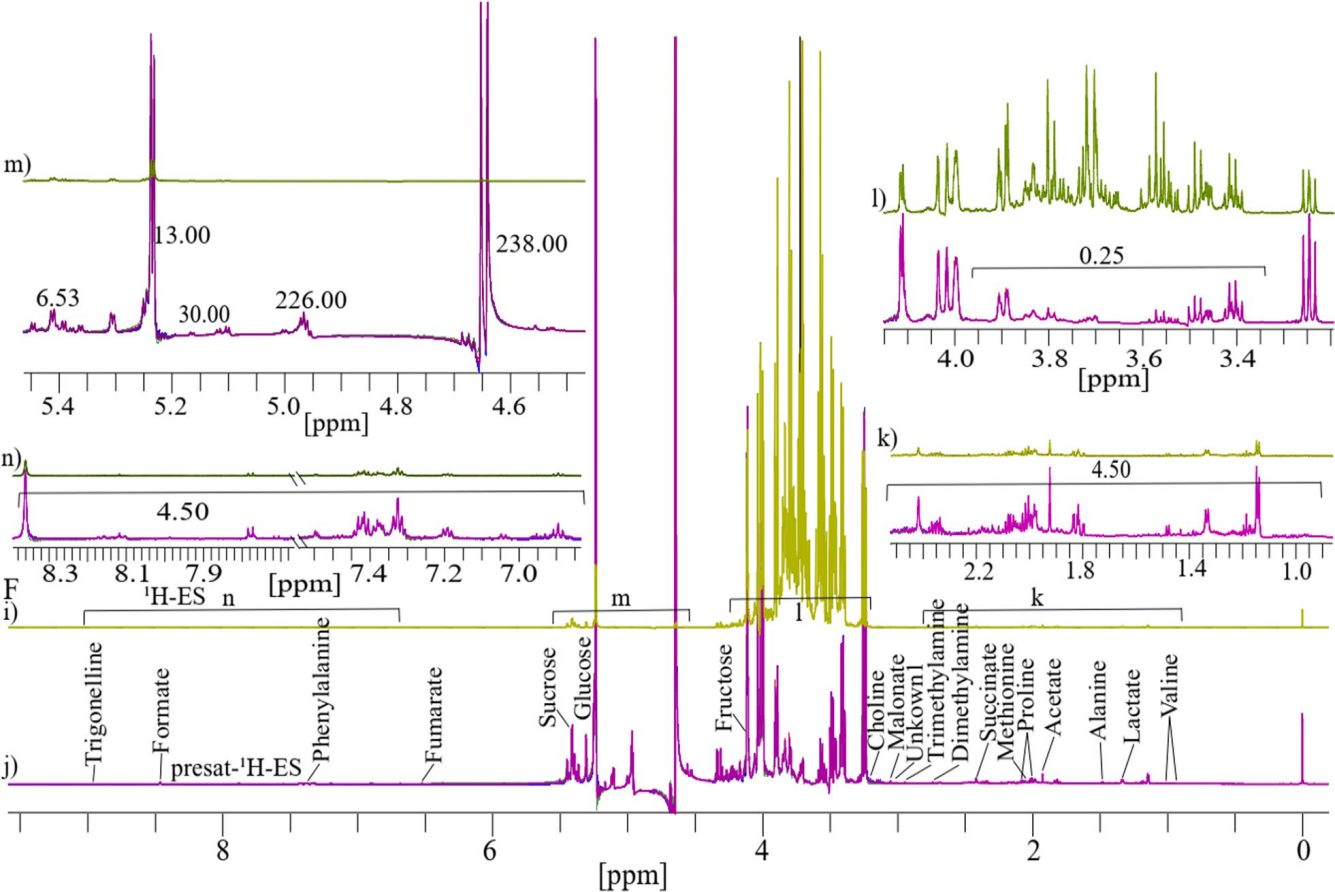
See **F** Typical 1D ^1^H-ES and presat-^1^H-ES NMR spectra comprising four replicate samples of honey dissolved in deuterium oxide, with TSP serving as a reference. These spectra were acquired using **a** ^1^H-ES and **b** presat-^1^H-ES, with metabolites assigned to the respective peaks

**Fig. 7:**
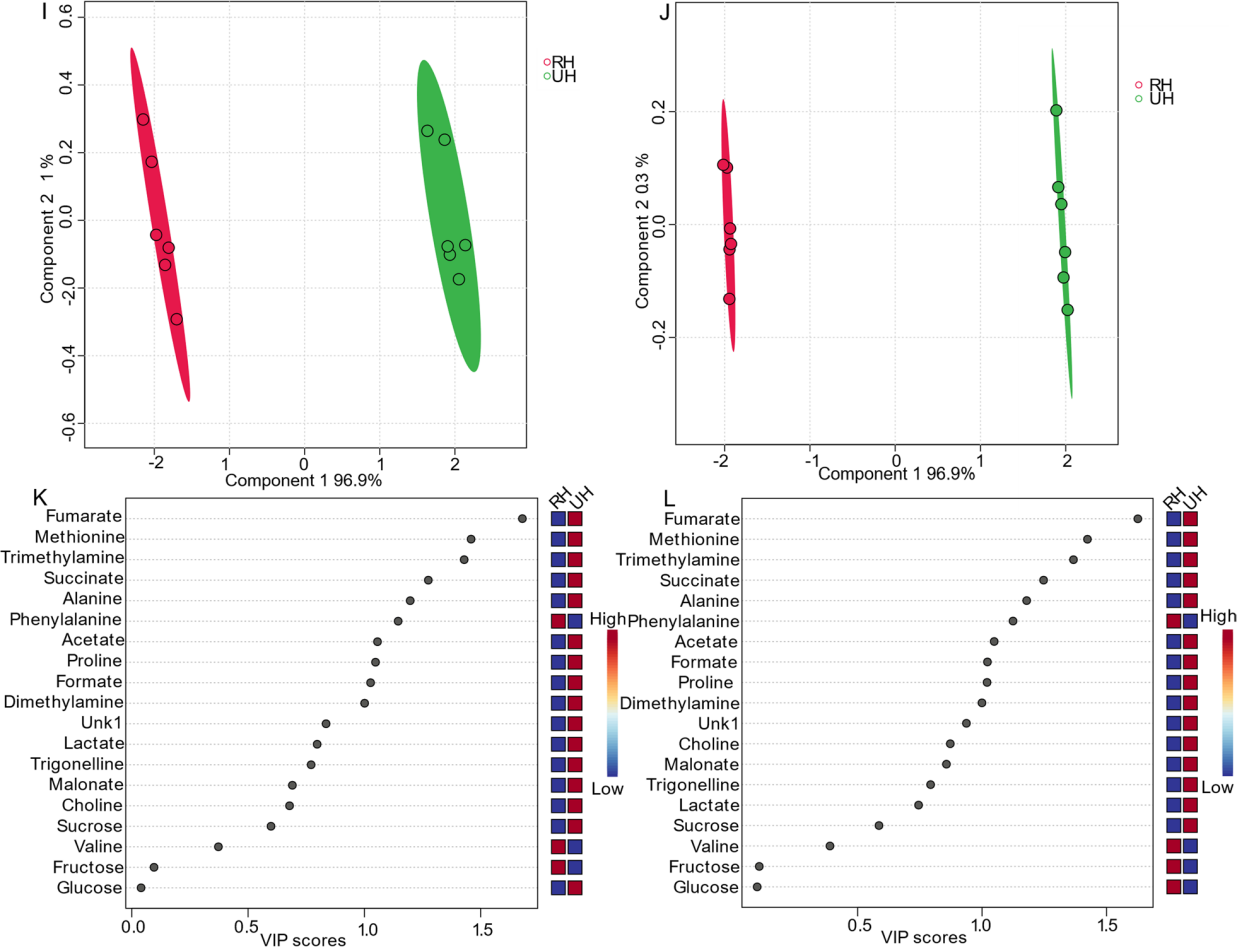
**i** PLS-DA based on metabolomics data from two types of honey samples, namely, RD (red, n = 6) and UD (green, n = 6), obtained using data from 1D ^1^H-ES NMR spectra. **j** PLS-DA score plot based on the same samples using data from 1D presat-^1^H-ES NMR spectra. The VIP score chart projection, calculated as the weighted sum of squares of the PLS-DA loadings considering the amount of explained Y-variation in each dimension, is based on data acquired from **k** 1D ^1^H-ES, and **l** presat-^1^H-ES NMR spectra

Similarly, as previously discussed, four replicates each of 1D ^1^H-ES and presat-^1^H-ES NMR spectra of honey are presented in Fig. 6F. The ^1^H-ES spectrum is also presented in Fig. 6i, and the presat-^1^H-ES spectrum is presented in Fig. 6j. Presat-^1^H-ES suppressed the sugar region while simultaneously enhancing the signal intensities of desired molecules. In the resulting presat-^1^H-ES spectrum (Fig. 6j), seventeen metabolites were selectively assigned across the full spectrum, demonstrating an improvement over the conventional ^1^H-ES method (Fig. 6i). Each full spectrum was segmented into four distinct sections that were further expanded to highlight specific chemical shift regions, namely, 0.76–2.61 (k), 3.20–4.15 (l), 4.55–5.45 (m), and 6.85–8.49 ppm (n). Across the entire spectrum, seventeen signals corresponding to identified and unidentified metabolites were chosen for quantification. This selection facilitated a comparative analysis between the spectra obtained using both NMR methods, permitting a comprehensive assessment of their performance. The full spectra were divided into four different sections indicated by k, l, m, and n. The suppression of the sugar region led to a remarkable 4.50-fold enhancement of peak intensities within the range of 0.76–2.61 ppm in the presat-^1^H-ES spectrum compared with the ^1^H-ES spectrum (Fig. 6k). Meanwhile, the residual signals from the suppressed sugars in the region of 3.40–3.90 ppm were reduced to 0.25-fold of that in the original method (Fig. 6l). In particular, the intensities of the β-glucose, α-glucose, and sucrose signals at 4.60, 5.20, and 5.40 ppm, respectively, were increased by 238.0, 13.0-, and 6.53-fold, respectively (Fig. 6m). In addition, the peak intensities in the region of 6.80–8.51 ppm were increased by 4.50-fold (Fig. 6n). Data from both ^1^H-ES and presat-^1^H-ES, consisting of twelve replicates from two distinct groups of honey (RH and UH), were subjected to multivariate analysis, including score plots, VIP scores, heatmaps, and cross-validation using PLS-DA. The 2D score plots derived from PLS-DA revealed significant separation between the two groups and notable clustering among replicates within the same group for honey samples analyzed using both 1D ^1^H-ES and presat-^1^H-ES (Fig. 7I, j, respectively). Comparison of visual score plots of both NMR methods show almost similar separation and clustering of groups whereas the method (presat-^1^H-ES) vs the method (^1^H-ES) shows a slightly greater collection of score points in component one (97.2%) vs (97.9%), and in the case of component two (1.3%) vs (0.3%). The VIP score plots, derived from the two components of PLS‐DA, were employed to identify the metabolites responsible for distinguishing between the RH and UH groups. These plots revealed that fumarate, methionine, trimethylamine, succinate, alanine, phenylalanine, acetate, formate, proline, and dimethylamine, each with VIP scores exceeding one, exhibited differences in abundance between the UH and RH groups, following the same pattern of the data obtained from both ^1^H-ES (Fig. 7k) and presat-^1^H-ES (Fig. 7l). These ten metabolites significantly contributed to the observed variation between the two groups. The remaining metabolites exhibited consistent VIP scores between both NMR methods, indicating the reproducibility of data analysis between presat-^1^H-ES and ^1^H-ES.

Heatmaps were generated to visualize the metabolomic profiles [45] derived from twelve NMR spectra obtained from the RH and UH groups. These heatmaps were constructed using data acquired via both 1D ^1^H-ES (Figure S5A) and 1D presat-^1^H-ES (Figure S5B). Upon comparison, the results revealed a high degree of similarity between the heatmaps generated by the two NMR methods, with the only difference being a reversal of their patterns. Interestingly, the heatmap derived from presat-^1^H-ES data revealed more homogeneous clustering among replicates within the same group than that derived from ^1^H-ES data. This enhanced clustering is attributable to the enhancement of peak intensities, which facilitated more accurate annotation within various metabolic processing software platforms.

We applied LOOCV to the ^1^H-ES (Figure S6A) and presat-^1^H-ES data (Figure S6B). Q2 was evaluated on five components, resulting in the following values: Q2 = 0.99881, R2 = 0.99986, and accuracy = 1.0 for ^1^H-ES (Figure S6C) and Q2 = 0.99978, R2 = 0.99991, and accuracy = 1.00 for presat-^1^H-ES (Figure S6D). These data demonstrated the comparable robustness of both methods. Notably, Q2 and R2 were significantly higher in the presat-^1^H-ES data than in the ^1^H-ES data owing to the substantial 4.50-fold enhancement of the desired signals while suppressing the dominant sugar signals. This enhancement significantly the accuracy of signal annotation processed using Chenomx software, leading to more precise and reliable results.

### Validation of the 1D presat-^1^H-ES NMR method and its comparison with 1D ^1^H-ES in agriculturally cultivated ajwa date of three different stages

**Fig. 8:**
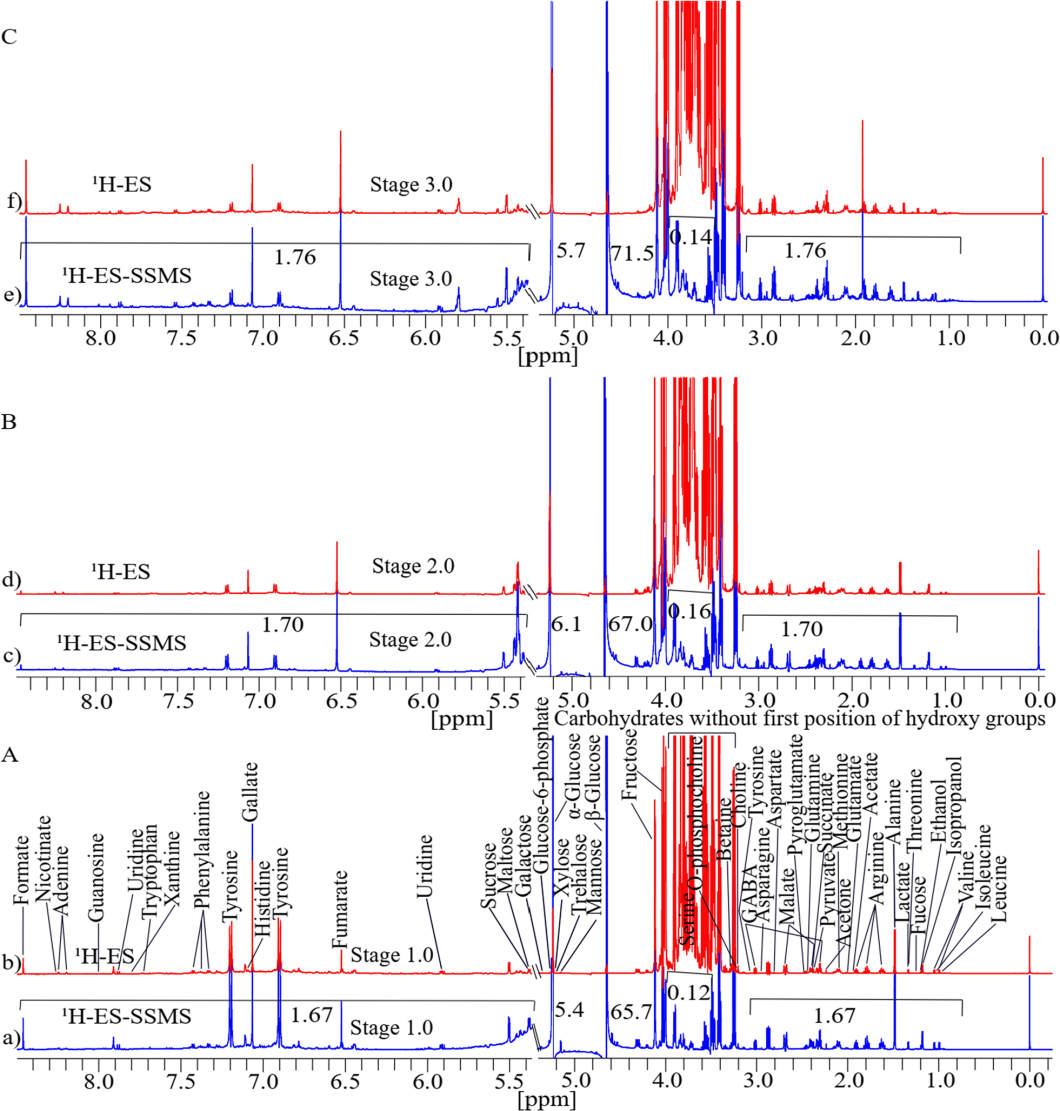
Typical 1D ^1^H NMR spectra of date flesh samples obtained at three different stages of growth, dissolved in deuterium oxide, with TSP as a reference. These spectra were acquired using ^1^H-ES and presat-^1^H-ES, with metabolites assigned to the respective peaks. Spectra for the first stage are presented in **a** for ^1^H-ES and **b** for presat-^1^H-ES in **A**; spectra for the second stage are presented in **c** for ^1^H-ES and **d** for presat-^1^H-ES in **B**; and spectra for the third stage are presented in **e** ^1^H-ES and **f** presat-^1^H-ES in **C**. Across the spectra, fifty peaks corresponding to the identified metabolites are presented

The identification of metabolites, comparison of their signal intensities, and statistical analysis of agricultural ajwa dates cultivated at three different stages (Khalal [half-ripe], Rutab [soft-ripened], and Tamr [fully ripened]) were cultivated with different time intervals into six stages, such as RZ1, RZ2, RZ3, RZ4, RZ5, and RZ6 (Table S1) to validate the presat-^1^H-ES NMR method in comparison to the ^1^H-ES NMR method. Figure 8A presents the spectra of both NMR methods for the first stage of date growth, divided into section (a) for ^1^H-ES and section (b) for presat-^1^H-ES. Similarly, the second stage is depicted in Fig. 8B, with section (c) representing ^1^H-ES and section (d) representing presat-^1^H-ES. The third stage is illustrated in Fig. 8C, with section (e) representing ^1^H-ES and section (f) representing presat-^1^H-ES.

The suppression of the sugar region led to a remarkable enhancement of peak intensities in the spectra obtained by presat-^1^H-ES compared with those obtained by ^1^H-ES, estimated as 1.67-fold for first stage samples (Fig. 8a), 1.70-fold for second stage samples (Fig. 8c), and 1.76-fold for third stage samples (Fig. 8e). Meanwhile, the residual signals from the suppressed sugar region of 3.40–3.90 ppm were reduced to 0.12-, 0.16-, and 0.14-fold in the first, second, and third stages, respectively (Fig. 8a, b, c). The peak intensities of β-glucose and α-glucose signals samples at 4.60 and 5.23 ppm, respectively, increased by 65.7- and 5.4-fold, respectively for the first stage (Fig. 8a), 67.0- and 6.1-fold, respectively, for the second stage (Fig. 8c), and 71.5- and 5.7-fold, respectively, for the third stage (Fig. 8e).

**Fig. 9:**
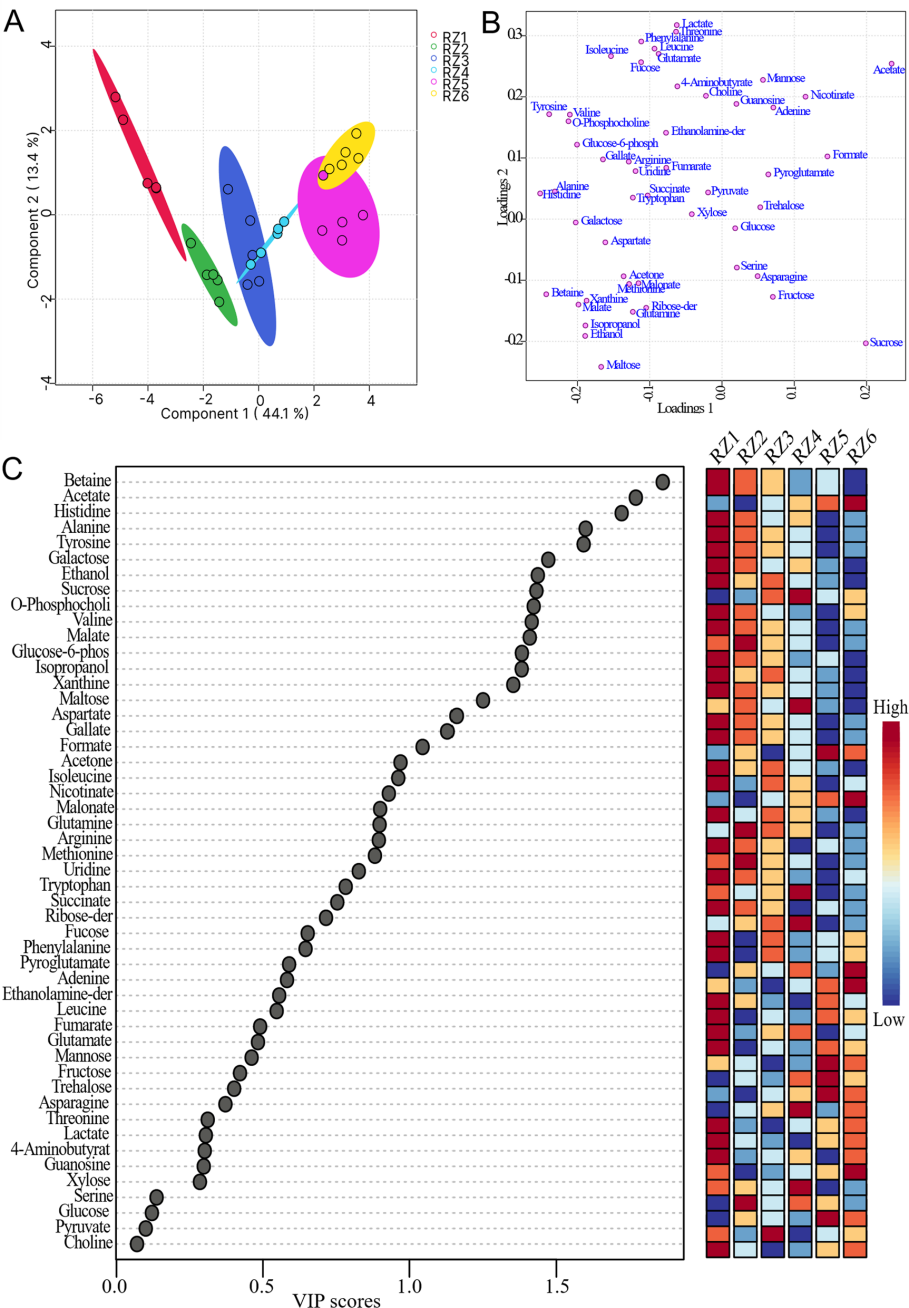
**a** PLS-DA based on metabolomics data distinguished the RZ1, RZ2, RZ3, RZ4, RZ5, and RZ6 groups, each presented with five replicates. **b** The loading plot generated from PLS-DA and **c** VIP score chart projection. This analysis used data derived from 1D presat-^1^H-ES NMR spectra

**Fig. 10:**
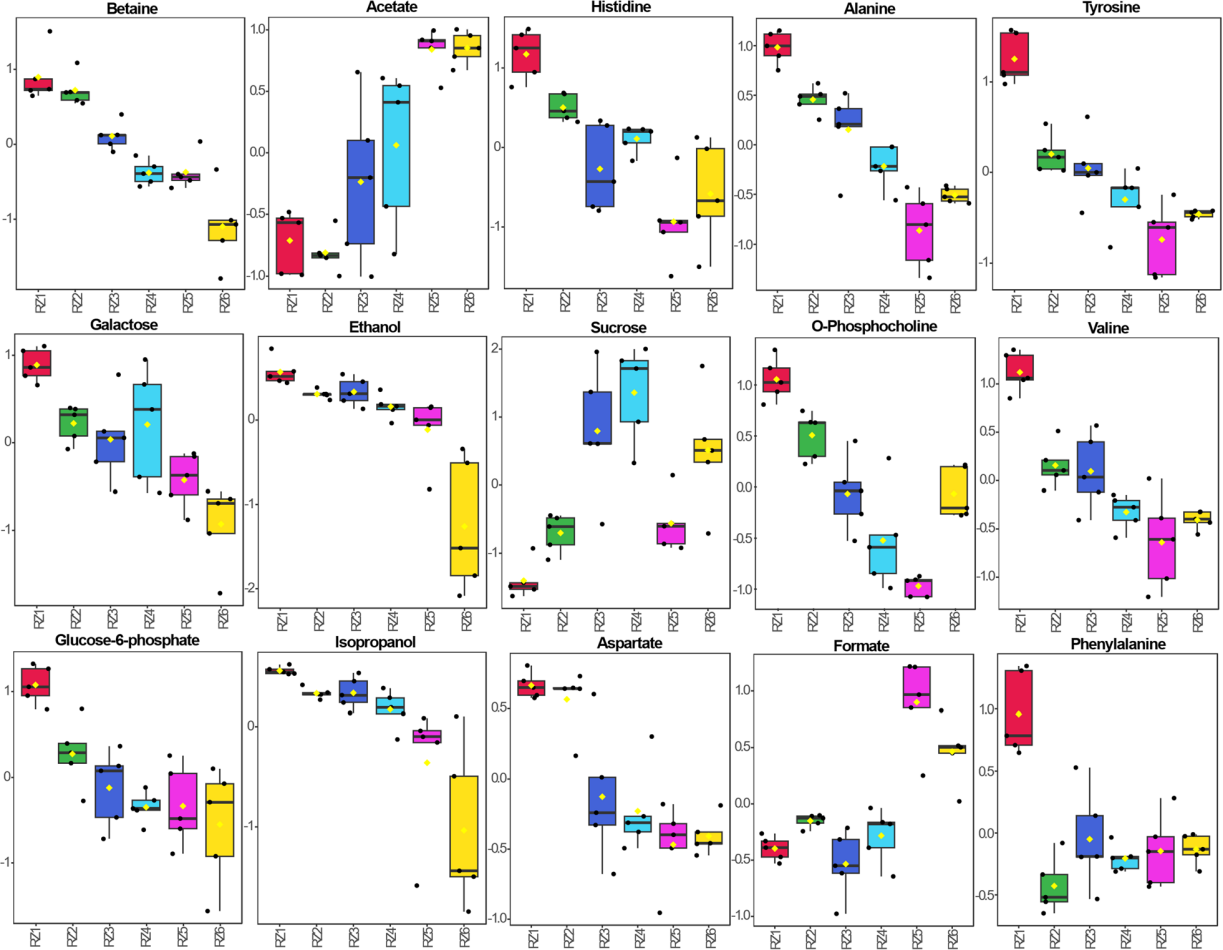
Boxplots of the concentrations of significant metabolites among the RZ1, RZ2, RZ3, RZ4, RZ5, and RZ6 groups for different stages of dates. Black dots represent outliers

The 1D ^1^H NMR spectra of five replicates of samples from six different stages (RZ1, RZ2, RZ3, RZ4, RZ5, and RZ6) representing three different stages of date flesh samples were subjected to multivariate analysis, including score plots, VIP scores, heatmaps, and cross-validation using PLS-DA for both the presat-^1^H-ES and ^1^H-ES NMR methods. The 2D score plots generated using PLS-DA demonstrated a clear separation among these six groups and notable clustering of five replicates within each group using data from the 1D presat-^1^H-ES method (Fig. 9a). The five replicates within each group exhibited significant clustering, indicating high reproducibility and consistent variation based on the concentration properties. This significant clustering reflects the model’s ability to effectively discriminate between the groups, suggesting that concentration variations are key drivers in differentiating these groups in the dataset. The loading plot generated using PLS-DA illustrates the contribution of each metabolite to the concentration that differentiates these groups (Fig. 9b). The loading plot presents the spreading of the maximum number of metabolites over the plot, which predicted a smaller number of metabolites with high variation of concentration among the groups. Eighteen metabolites with VIP > 1.0 (common cutoff) were considered significant contributors to the six-group separation (Fig. 9c). The VIP plot identified five metabolites (betaine, acetate, histidine, alanine, and tyrosine) with a VIP score of > 1.5 as critical discriminators of the groups. The box and whisker plot analysis revealed clear distinctions in metabolite abundance distributions among the six date flesh groups (Fig. 10). For betaine, RZ1 displayed extended upper whiskers, indicative of a high-abundance profile, whereas the lowest whisker in RZ6 reflected that this profile had the lowest abundance. The lowest whisker for acetate in RZ6 denoted the lowest abundance of this profile. The RZ3 and RZ4 groups displayed the broadest interquartile range and extended moderate upper whiskers, indicative of high variability and a right-skewed abundance profile, whereas the highest whisker in RZ5 reflected the highest abundance among the groups. The abundance of histidine was the highest in the RZ1 group, followed by the RZ2, RZ4, RZ3, RZ6, and RZ5 groups. The important essential amino acid tyrosine exhibited the highest abundance in RZ1, followed by RZ2, RZ3, RZ4, RZ6, and RZ5. The abundance of aspartate is the highest in the RZ1 and RZ2 groups, followed by the RZ3, RZ4, RZ5, and RZ6 groups. The abundance of sucrose was the highest in RZ4, with significant variability within this group, followed by RZ3, RZ6, RZ5, RZ2, and RZ1. Aspartate has abundance in higher order RZ1, RZ2, RZ3, RZ4, RZ5, and RZ6 groups. Phenylalanine has a lower value of VIP than the significant value of VIP score, but it is an important essential amino acid that is found in order of highest abundance in RZ1, and lower in order of RZ5, RZ6, RZ3, RZ4, and RZ2 groups. The abundance of other metabolites is described similarly.

A comparison of the visual score plots from both ^1^H-ES and presat-^1^H-ES NMR revealed a high degree of similarity in group separation and clustering. There were small differences in values of the components of scores in the 2D plots of PLS-DA for the separation study among the six different groups of date flesh analyzed by ^1^H-ES and presat-^1^H-ES. Comparisons of values for ^1^H-ES and presat-^1^H-ES in components one (42.3% vs. 44.1%) and two (15.6% vs. 13.4%) are presented in Figure S7A and S7B, respectively. The VIP score plots, derived from two components of PLS‐DA, were employed to identify metabolites distinguishing the groups, and a comparison of abundance using data obtained by ^1^H-ES and presat-^1^H-ES is presented in Figure S7C and S7D, respectively. The metabolites exhibited consistent VIP scores across both NMR methods, indicating the similar reproducibility of data analysis between presat-^1^H-ES and ^1^H-ES.

Heatmaps were generated to visualize the metabolomic profiles derived from NMR spectra originating from groups representing the three distinct stages of growth. These heatmaps were constructed using data acquired by both 1D ^1^H-ES (Figure S8A) and 1D presat-^1^H-ES (Figure S8B). Upon comparison, the results revealed a high degree of similarity between the heatmaps generated using data from both NMR methods, with the only difference being higher clustering in the profiling of data from 1D presat-^1^H-ES. This enhanced clustering can be attributed to the augmentation of peak intensities, which aids in more accurate annotation within various metabolic processing software platforms.

We employed LOOCV to data from both 1D ^1^H-ES (Figure S9A) and 1D presat-^1^H-ES NMR spectra (Figure S9B). Q2 was assessed over five components, yielding the following results: Q2 = 0.94415, R2 = 0.98275, and accuracy = 0.73333 for ^1^H-ES (Figure S9C) and Q2 = 0.93382, R2 = 0.96965, and accuracy = 0.76667 for presat-^1^H-ES (Figure S9D). These values indicate the development of a robust and reliable model using presat-^1^H-ES data. The data highlights the comparable robustness of the two NMR methods. Notably, Q2 and R2 were significantly higher for ^1^H-presat-ES than for ^1^H-ES method. This increment is attributable to an average 1.71-fold enhancement of signal intensities, which was achieved through the suppression of sugar signals. The natural abundance of metabolites in the different regions of functional groups was quantified in 1D ^1^H-ES NMR spectra relative to the TSP internal reference (Figure S10A), revealing the extremely high intense peaks in the region of functional groups of sugar compared with the findings in other important regions of functional groups of metabolites. Similarly, quantification in 1D presat-^1^H-ES NMR spectra after suppression of the extremely high-intensity peaks in the region of functional groups of sugar resulted in the enhancement of peaks of other important regions of functional groups (Figure S10B). This enhancement substantially improved the accuracy of signal annotation achieved using Chenomx software, resulting in more precise and reliable analytical outcomes.

The enhancement in peak intensities resulting from the suppression of sugar peaks with residual signals was substantial (0.15-, 0.12-, and 0.25-fold), resulting in 3.04-, 2.55-, and 4.50-fold increases in the intensity of desired peaks of date flesh, ripe mango flesh, and honey samples, respectively, in presat-^1^H-ES compared with ^1^H-ES. These enhancements in peak intensities increased the visibility of the desired peaks relative to sugar signals, thereby improving the reliability of the analytical data and reducing experimental time.

Multivariate analysis was performed through various techniques, such as score plots, VIP analysis, heatmaps, and cross-validation via PLS-DA, for both the presat-^1^H-ES and ^1^H-ES NMR methods. The 2D score plots derived from PLS-DA exhibited significant separation and clustering among replicates within the same groups for both groups of date flesh, ripe mango flesh, and honey samples, indicating the effectiveness of both methods in differentiating the different groups.

Multivariate analysis is widely used for metabolic profiling in NMR-based metabolomics, particularly in the analysis of various complex mixtures, such as plants [46–49], marine life [50, 51], and other biological samples [52–54]. This analytical approach aids in the identification of significant biomarkers pivotal for diagnostic purposes [55–57], and estimation of nutritional levels [57, 58].

Further analysis using VIP score plots highlighted metabolites in the samples while discriminating between the different groups. Notably, different metabolites in different samples displayed similar VIP scores in presat-^1^H-ES and ^1^H-ES, indicating that the two NMR methods provide identical distinction among the groups. The heatmaps generated by both NMR methods demonstrated similarities in overall patterns, with the presat-^1^H-ES method exhibiting more homogeneous clustering within each group, likely because of enhanced peak intensities, which facilitated more precise metabolite annotation.

The consistency and reproducibility of the results were confirmed through cross-validation, which demonstrated the robustness of the models constructed using data from both the conventional and modified NMR methods. High Q2 and R2 indicated excellent predictive accuracy and model fitting, reinforcing the level of presat-^1^H-ES for metabolomics studies. Comparisons between the conventional ^1^H-ES method and the modified presat-^1^H-ES method across different types of samples, including date flesh, mango flesh, and honey, illustrated that presat-^1^H-ES consistently achieved increments in sensitivity, reproducibility, and reliability in this metabolomics study. This highlights the versatility and applicability of the proposed enhancement across various sample matrices, underscoring its potential for widespread adoption in NMR-based metabolomics research [59–61].

The presat-^1^H-ES methodology offers distinct advantages over conventional SSMS techniques for routine metabolomics analyses. Previous suppression methods (e.g., SSMS) suffer from long pulses and calibration burdens that reduce throughput and introduce relaxation and lineshape artifacts near water and crowded regions. Presat‑^1^H‑ES overcomes these limits: its Sinc1.1000 shaped pulse is more than three times shorter than the EBurp1 used in SSMS, yielding a sharper excitation bandwidth, reduced relaxation-induced distortion, and improved quantitative/qualitative signal integrity [62], making it better suited for routine metabolomics. However, the presat-^1^H-ES method is hampered by its dephasing of highly intense peaks of sugar in the region of 4.55–5.45 ppm, but it is suitable for binning in AMIX [63] and annotation in Chenomx [64] for the comparative quantification of metabolites. The 4.55–5.45 ppm region will be used to compare the concentrations of mono- and disaccharide carbohydrates by the presat-^1^H-ES method. It is suitable only for samples such as dates, honey, rice, dairy products, and sweet fruits and juices, which have incomparably high sugar levels compared with other metabolites. Regarding other drawbacks of presat-^1^H-ES, unless it is calibrated with appropriate internal standards to calculate its response factors, this method is not suitable for absolute quantification.

Significant benefits for applications involving food quality were provided by the effective optimization of the 1D presat-^1^H-ES NMR approach for metabolomic profiling of date palm fruits maturation across ripening stages (Khalal, Rutab, and Tamr). This technique increased the detection of nutritionally significant metabolites by suppressing prominent sugar signals. It also revealed ripening-dependent variations in betaine (stress response) [65], sucrose (sweetness) [66], and amino acids (flavor precursors) [67]. Precise profiling of quality indicators was made possible by the increased sensitivity (1.67–1.76 factors): late-stage acetate buildup suggested fermentation, mid-ripening peaks in sucrose associated with ideal sweetness, and early-stage dates displayed strong betaine (osmoprotectant). A foundation for objective ripeness categorization was established by PLS-DA models, which discovered VIP > 1.0 discriminators (betaine, acetate, histidine, alanine, and tyrosine).

## Conclusion

In summary, this study developed 1D presat-^1^H-ES NMR as a novel approach for metabolomics studies by selectively suppressing the dominant sugar signals and enhancing the signal intensity of low‑abundance metabolites. The results demonstrated significant increments in the visibility and signal intensity of low-concentration target metabolites, which were achieved through the effective suppression of overwhelming signals from dominant, abundant sugars. This advancement could facilitate the superior identification and relative quantification of both desired metabolites and sugars in metabolomics research. Consequently, this methodology provides more precise and dependable analyses while substantially reducing the experimental duration required to acquire NMR spectra in comparison with the conventional 1D ^1^H-ES NMR method for complex samples, particularly those dominated by high sugar concentrations relative to other metabolites. The 1D presat-^1^H-ES NMR method was validated through the analysis of date flesh samples at three ripening stages; this method outperformed conventional 1D ^1^H-ES NMR by suppressing the dominant sugar signals (3.40–3.90 ppm) and significantly enhancing the signal intensities of desired metabolites. The identification of fifty metabolites and multivariate analysis (PLS-DA, VIP scores, and heatmaps) confirmed superior group separation in presat-^1^H-ES. Betaine, acetate, histidine, alanine, tyrosine, galactose, ethanol, sucrose, o-phosphocholine, valine, glucose-6-phosphate-der, isopropanol, aspartate, formate, and phenylalanine were identified as key metabolites for the characterization of the nutritional content of samples. In the date sector, where market value is determined by sugar content and taste development, these results show how presat-^1^H-ES NMR may track postharvest biochemical changes to harvest timing, processing, and shelf-life. In order to create predicted quality models, future research could connect presat-^1^H-ES NMR characteristics with sensory evaluation. The new method also demonstrated higher accuracy, reliability, and reproducibility, and broader applicability in sugar-rich samples, improving both speed and precision in metabolomics. Consequently, this modified approach offers substantial promises for applications in food quality assessment, biomarker discovery, and comprehensive metabolic profiling in agricultural and nutritional research.

## Supplementary Information

Supplementary material 1. Figures S1 to S10 and Table S1 include NMR-based metabolomics heatmap analyses, PLS-DA cross-validation charts, score plots, and column charts for date flesh, ripe mango flesh, and honey samples, along with cultivation data for dates at different growth stages and a pulse program for presat-^1^H-ES.

## Data Availability

The data supporting this article have been included as part of the Supporting Information.
