# Supplementary material for: Enhancing detection of low‑abundance metabolites in proton NMR through band‑selective suppression and presaturation
Source: Nat Prod Bioprospect. 2026 Jan 11;16(1):16. doi: 10.1007/s13659-025-00570-3 (PMC12790556; doi:10.1007/s13659-025-00570-3)
Supplement: Supplementary file 1 — Supplementary material 1. Figures S1 to S10 and Table S1 include NMR-based metabolomics heatmap analyses, PLS-DA cross-validation charts, score plots, and column charts for date flesh, ripe mango flesh, and honey samples, along with cultivation data for dates at different growth stages and a pulse program for presat-1H-ES. [file 13659_2025_570_MOESM1_ESM.docx]

***Supporting Information***

**Enhancing detection of low‑abundance metabolites in proton NMR through band‑selective suppression and presaturation**

Upendra Singh¹, Renad Z. Al Ahmadi², Ruba Al‑Nemi², Manel Dhahri³, Mohammed S. Alarawi⁴, Abdul Aziz^5^, Faisal Abdulaziz Bushulaybi^6^, Tamer Abdalla Mashtoly^7^,

Abdul‑Hamid Emwas^8,^*, Lukasz Jaremko¹^,^*, and Mariusz Jaremko^9,^*

¹ Department of Biochemistry & Molecular Biology (BMB), Sealy Institute for Drug Discovery (SIDD), University of Texas Medical Branch (UTMB), Galveston, TX 77555‑1068, USA

² Division of Biological and Environmental Sciences and Engineering (BESE), King Abdullah University of Science and Technology (KAUST), Thuwal, Makkah 23955‑6900, Saudi Arabia

³ Department of Biology, College of Science, Taibah University, Yanbu Governorate, Saudi Arabia.

⁴ Computational Bioscience Research Center, KAUST, Thuwal, Makkah 23955‑6900, Saudi Arabia

^5^ National Center for Palms and Dates, Prince Turki ibn Abdulaziz Al Awwal Rd, 13512,

Hittin-Riyadh-Kingdom of Saudi Arabia.

^6^ WEQAA Center, Al-Rabwa, Riyadh 12813, Saudi Arabia

^7^ Plant protection Dept. Faculty of Agriculture, Ain Shams University, Cairo, Egypt . P.O 11241 Hadayek Shoubra

^8^ Core Lab of NMR, KAUST, Thuwal, Makkah 23955‑6900, Saudi Arabia

^9^ The Golden Ratio Institute, Riyadh 13244, Saudi Arabia

*Corresponding authors’ E‑mails:

[mariusz.jaremko@goldenratio.institute](mailto:mariusz.jaremko@goldenratio.institute) (M.J.); [abdelhamid.emwas@kaust.edu.sa](mailto:abdelhamid.emwas@kaust.edu.sa) (A.‑H.E.); [lukasz.jaremko@utmb.edu](mailto:lukasz.jaremko@utmb.edu) (L.J.)

**Contents:**

**Fig. S1. NMR-based metabolomics heatmap analysis of two types of date flesh samples.**

**Fig. S2. PLS-DA cross-validation charts based on NMR-based metabolomics between two types of date flesh samples.**

**Fig. S3. NMR-based metabolomics heatmap analysis of two types of ripe mango flesh samples.**

**Fig. S4. PLS-DA cross-validation charts based on NMR-based metabolomics between the two types of ripe mango flesh samples.**

**Fig. S5. NMR-based metabolomics heatmap analysis of two types of honey samples.**

**Fig. S6. PLS-DA cross-validation charts based on NMR-based metabolomics between the two types of honey samples.**

**Table S1.** **Cultivation of dates at different stages with different intervals of its growth.**

**Fig. S7. Score plots of partial least squares-discriminant analysis based on metabolomics data from six different types (RZ1, RZ2, RZ3, RZ4, RZ5, and RZ6) of samples with five replicates of each type.**

**Fig. S8. NMR-based metabolomics heatmap analysis of date flesh samples among three cultivation stages.**

**Fig. S9. PLS-DA cross-validation charts based on NMR-based metabolomics among date flesh samples at three stages of cultivation.**

**Fig. S10. Column charts of the ratios of the natural abundance of metabolites in date flesh samples at three stages of cultivation.**

**Pulse program of presat-^1^H-ES**


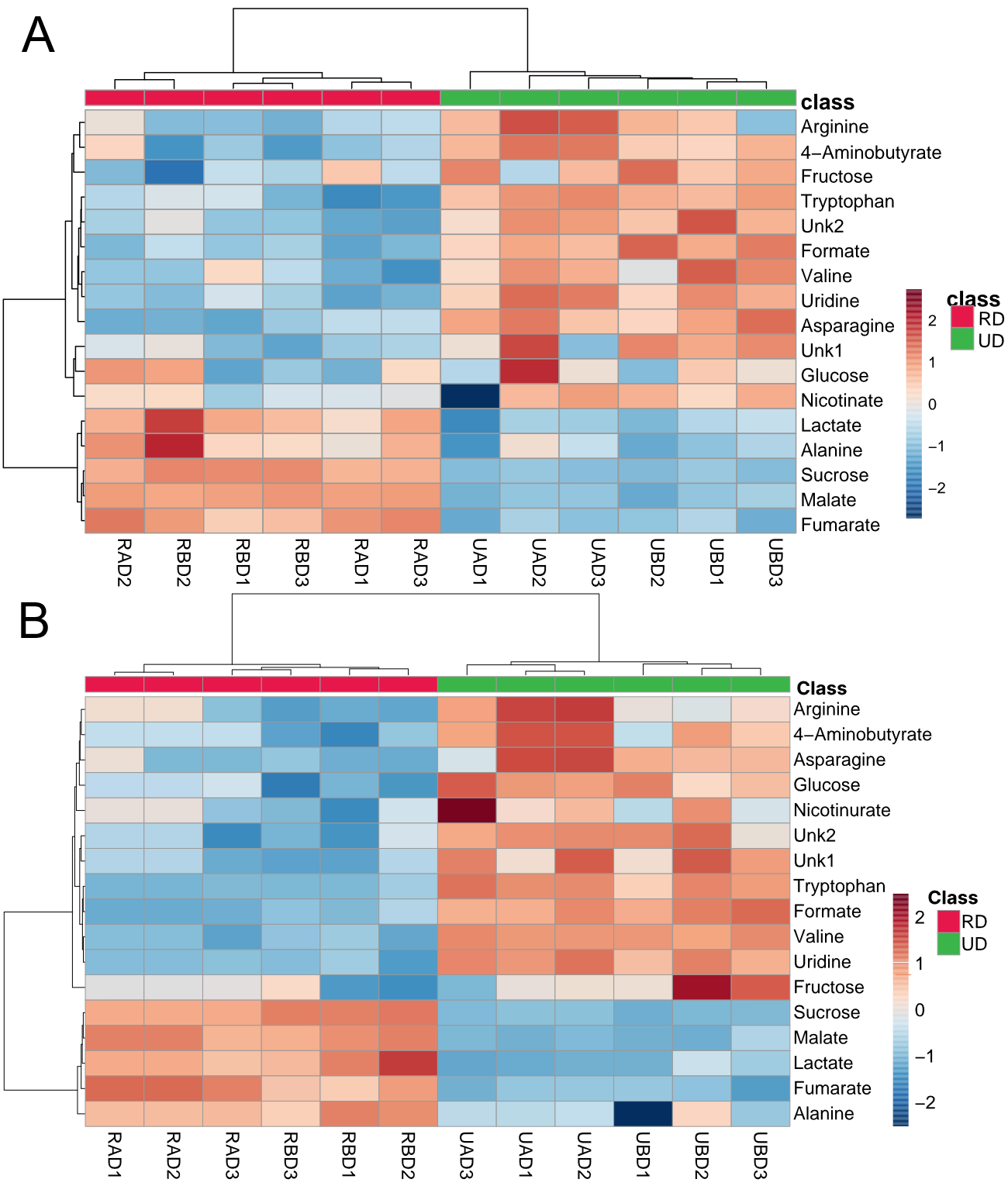


**Fig. S1.** NMR-based metabolomics heatmap analysis of two types of date flesh. The heatmaps are based on (A) 1D ^1^H-ES and (B) 1D presat-^1^H-ES spectra. The rows indicate the metabolites with their concentrations, whereas the columns present the replicates of samples extracted in a solution of methanol and water. Metabolites with decreased concentrations are presented in blue, whereas metabolites with increased concentrations are presented in red.


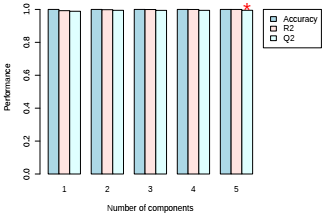

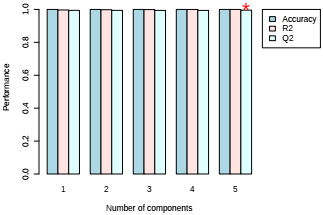

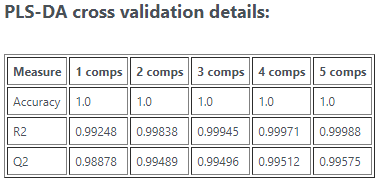

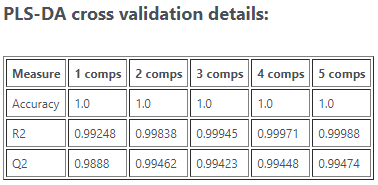


B

A

C

D

**Fig. S2.** PLS-DA cross-validation charts based on NMR-based metabolomics between the two types of date flesh. Data were acquired from (A) 1D ^1^H-ES and (B) 1D presat-^1^H-ES spectra. PLS-DA cross-validation details were obtained from (C) 1D ^1^H-ES and (D) 1D presat-^1^H-ES spectra.


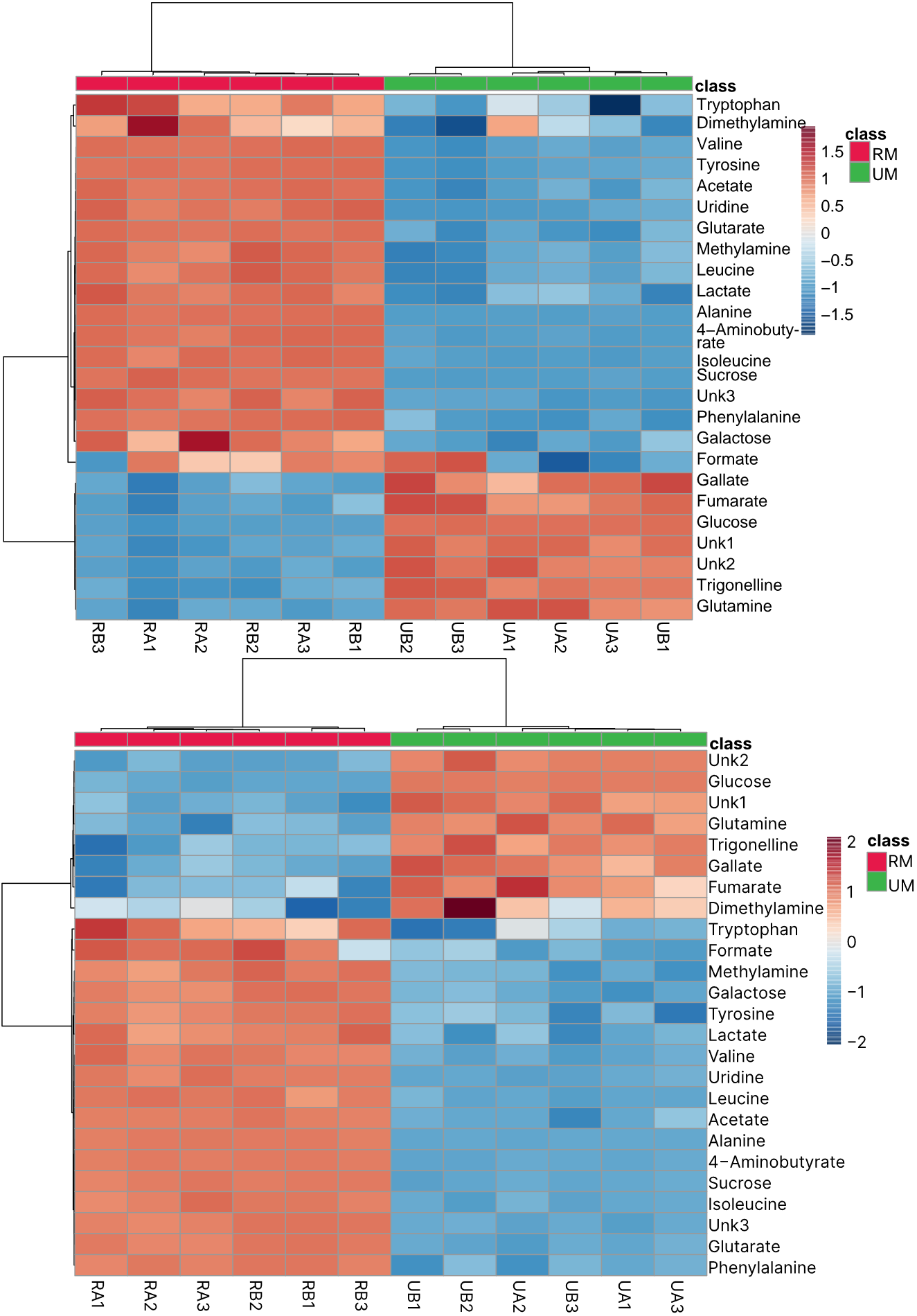


**Fig. S3.** NMR-based metabolomics heatmap analysis of two types of ripe mango flesh. Heatmaps are based on (A) 1D ^1^H-ES and (B) 1D presat-^1^H-ES spectra.


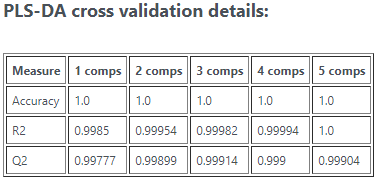

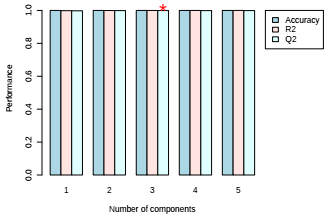

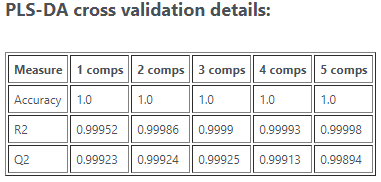

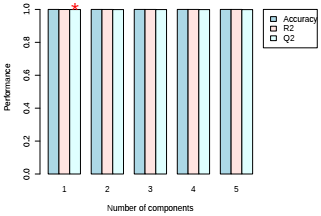


B

A

C

D

**Fig. S4.** PLS-DA cross-validation charts based on NMR-based metabolomics between the two types of ripe mango flesh. Data were acquired from (A) 1D ^1^H-ES and (B) 1D presat-^1^H-ES spectra. PLS-DA cross-validation details were obtained from (C) 1D ^1^H-ES and (D) 1D presat-^1^H-ES spectra.


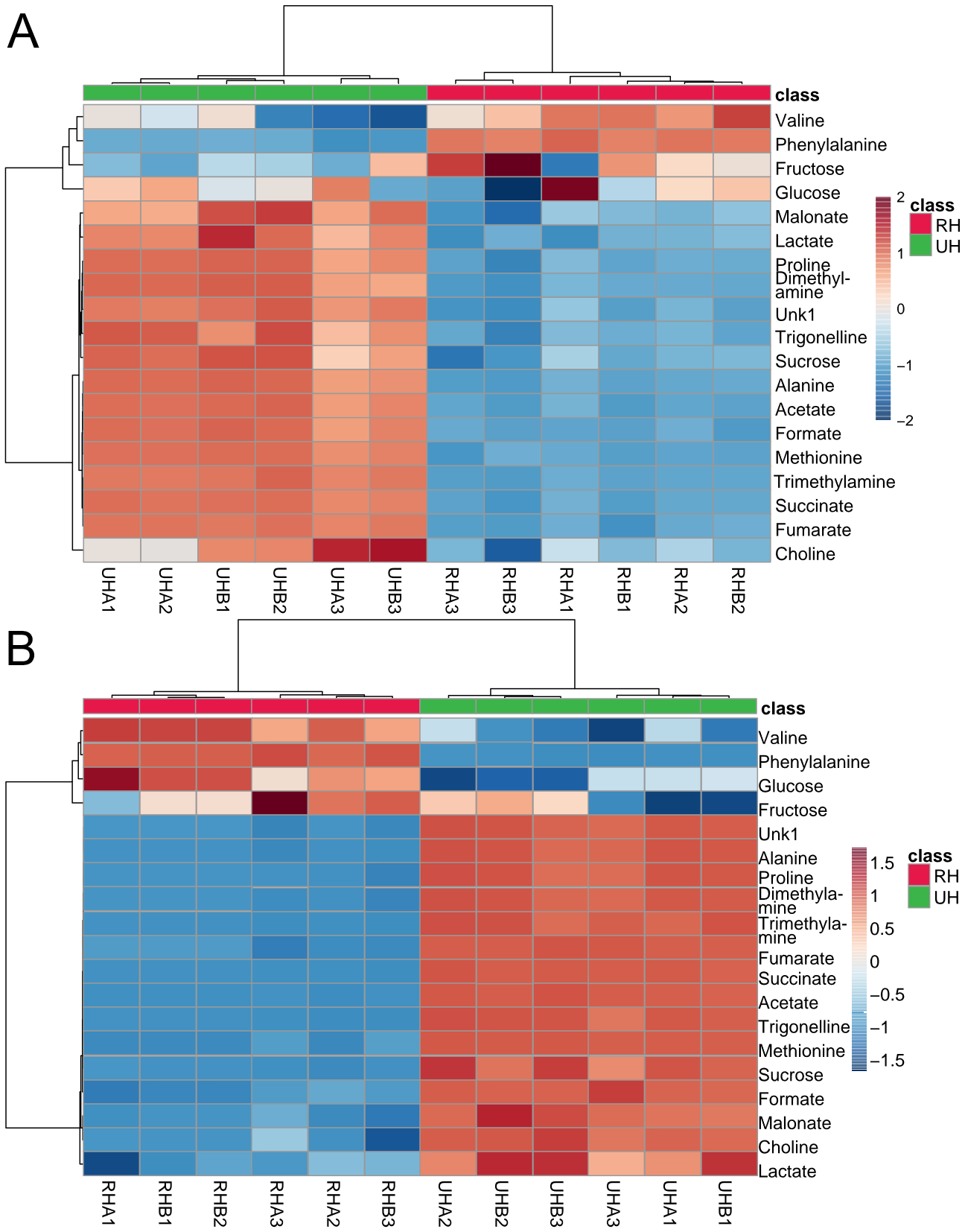
**Fig. S5.** NMR-based metabolomics heatmap analysis of two types of honey. The heatmaps are based on (A) 1D ^1^H-ES and (B) 1D presat-^1^H-ES spectra.


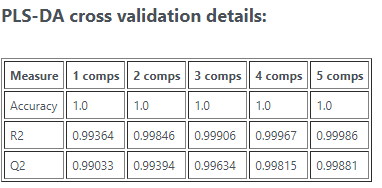

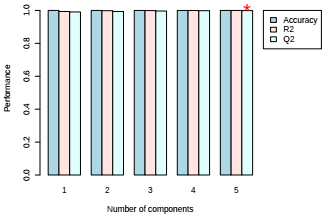

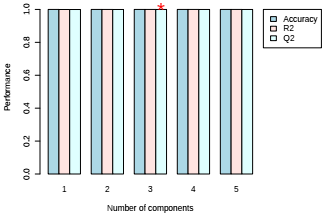

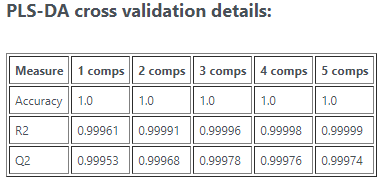


B

A

C

D

**Fig. S6.** PLS-DA cross-validation charts based on NMR-based metabolomics between the two types of honey. Data were acquired from (A) 1D ^1^H-ES and (B) 1D presat-^1^H-ES spectra. PLS-DA cross-validation details were obtained from (C) 1D ^1^H-ES and (D) 1D presat-^1^H-ES spectra.

| Date of cultivation of date | Stages |
| --- | --- |
| 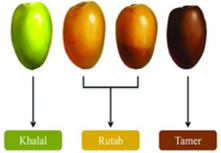Sample 1: Khalal stage, taken 10/06/2023  Sample 2: Khalal stage, taken 15/06/2023  Sample 3: Ruthab stage, taken 15/07/2023  Sample 4: Ruthab stage, taken 25/07/2023  Sample 5: Tamer stage, taken 15/08/2023  Sample 6: Tamer stage, taken 25/08/2023 | RZ1  RZ2  RZ3  RZ4  RZ5  RZ6 |

**Table S1.** Cultivation of dates at different stages with different intervals of their growth


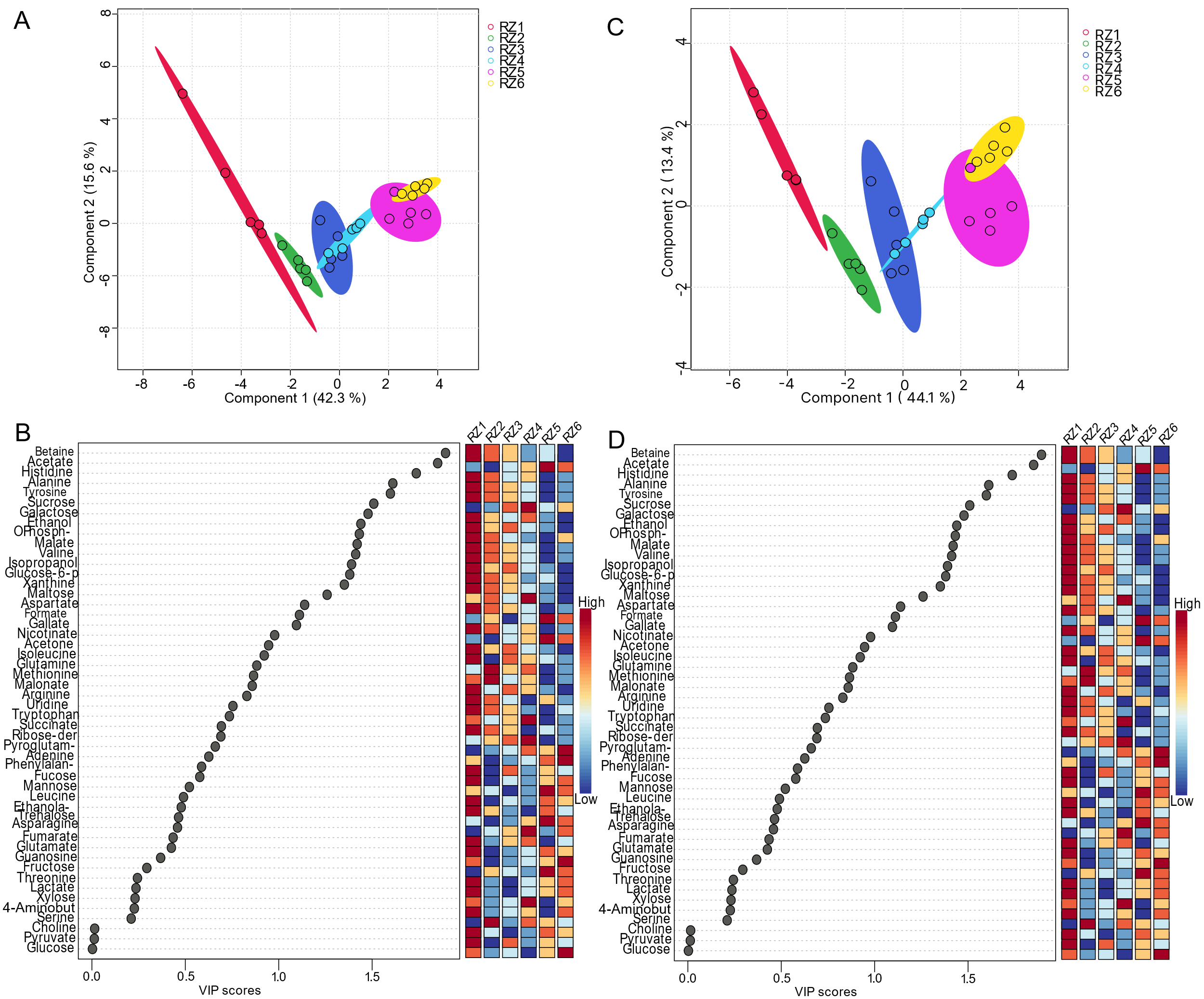


**Fig. S7.** Score plots of partial least squares-discriminant analysis (PLS-DA) based on metabolomics data from six different groups (RZ1, RZ2, RZ3, RZ4, RZ5, and RZ6) with five replicates of each type. Data was obtained from (A) 1D ^1^H-ES and (B) 1D presat-^1^H-ES NMR spectra. Variable importance of (VIP) score chart projection from data acquired from (C) 1D ^1^H-ES and (D) 1D presat-^1^H-ES NMR spectra.


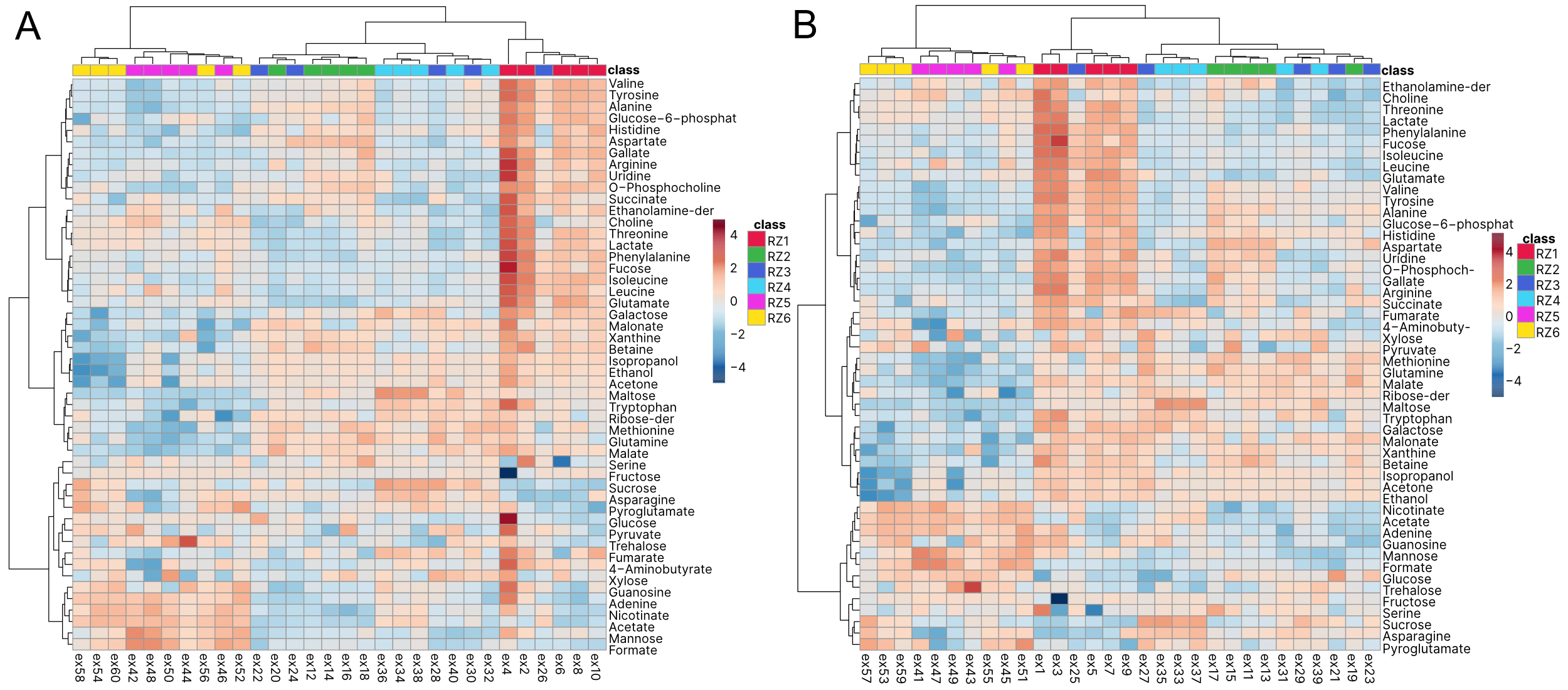


**Fig. S8.** NMR-based metabolomics heatmap analysis of date flesh among three cultivation stages. The heatmaps are based on (A) 1D ^1^H-ES and (B) 1D presat-^1^H-ES spectra.


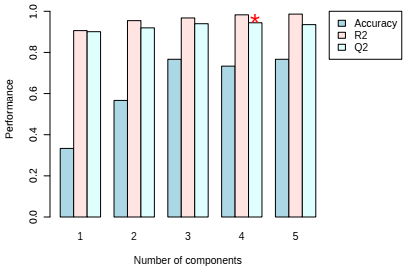

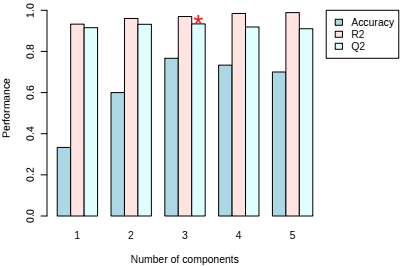

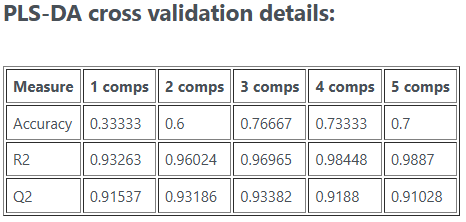

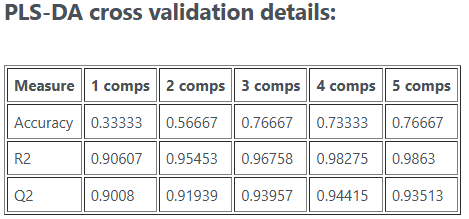


B

A

D

C

**Fig. S9.** PLS-DA cross-validation charts based on NMR-based metabolomics among date flesh from six different groups. Data were acquired from (A) 1D ^1^H-ES and (B) 1D presat-^1^H-ES spectra. PLS-DA cross-validation details were obtained from (C) 1D ^1^H-ES and (D) 1D presat-^1^H-ES spectra.

**Fig. S10.** Column charts of the ratios of the natural abundance of metabolites in date flesh at three stages of cultivation. Data were obtained using (A) 1D ^1^H-ES and (B) presat-^1^H-ES spectra.


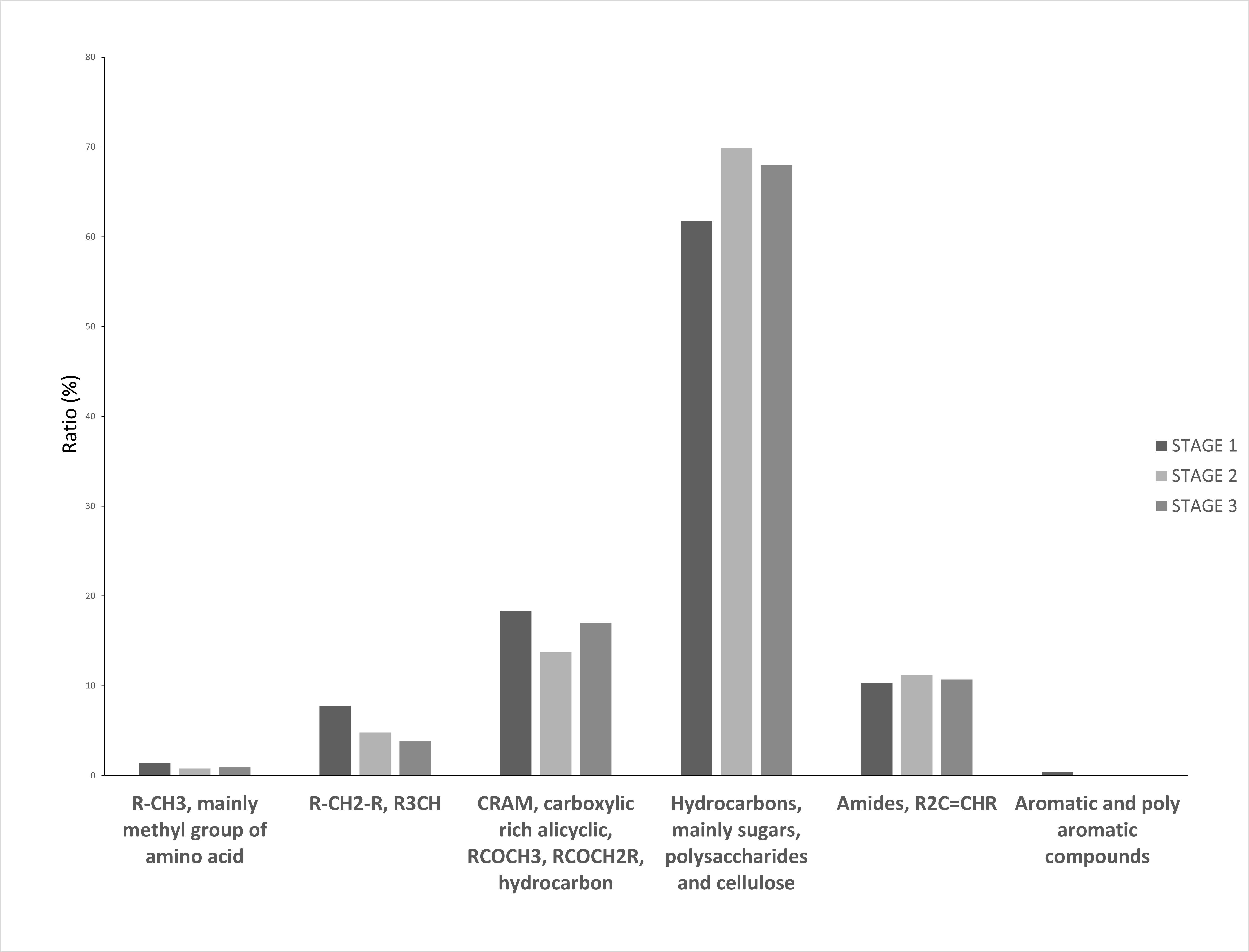

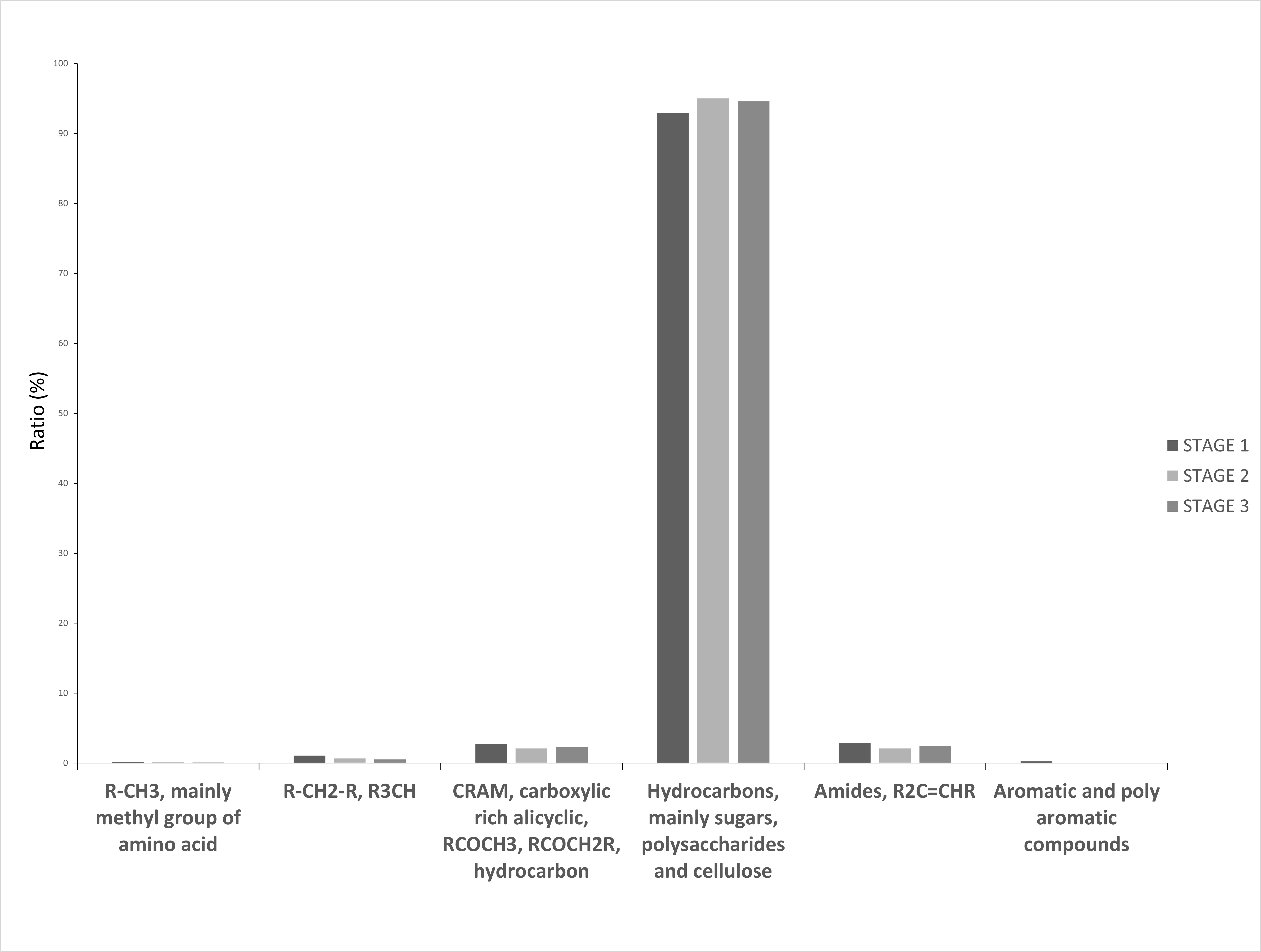


B

A

**Pulse program of presat-^1^H-ES**

;zgesgp_supp

;avance-version (12/01/11)

;1D sequence

;water suppression using excitation sculpting with gradients

;T.-L. Hwang & A.J. Shaka, J. Magn. Reson.,

; Series A 112 275-279 (1995)

;

;$CLASS=HighRes

;$DIM=1D

;$TYPE=

;$SUBTYPE=

;$COMMENT=

prosol relations=<triple>

#include <Avance.incl>

#include <Grad.incl>

#include <Delay.incl>

"p2=p1*2"

"d12=20u"

"TAU=de+p1*2/3.1416+50u"

"acqt0=0"

baseopt_echo

1 ze

2 30m

d12 pl9:f1

d1 cw:f1 ph29

4u do:f1

d12 pl1:f1 BLKGRAD

p1 ph1

50u UNBLKGRAD

p16:gp1

d16 pl0:f1

(p12:sp1 ph2:r):f1

4u

d12 pl1:f1

p2 ph3

4u

p16:gp1

d16

TAU

p16:gp2

d16 pl0:f1

(p12:sp1 ph4:r):f1

4u

d12 pl1:f1

p2 ph5

4u

p16:gp2

d16

go=2 ph31

30m mc #0 to 2 F0(zd)

4u BLKGRAD

exit

ph1=0

ph2=0 1

ph3=2 3

ph4=0 0 1 1

ph5=2 2 3 3

ph29=0

ph31=0 2 2 0

;pl0 : 0W

;pl1 : f1 channel - power level for pulse (default)

;sp1 : f1 channel - shaped pulse 180 degree

;p1 : f1 channel - 90 degree high power pulse

;p2 : f1 channel - 180 degree high power pulse

;p12: f1 channel - 180 degree shaped pulse (Squa100.1000) [2 msec]

;p16: homospoil/gradient pulse

;d1 : relaxation delay; 1-5 * T1

;d12: delay for power switching [20 usec]

;d16: delay for homospoil/gradient recovery

;ns: 8 * n, total number of scans: NS * TD0

;ds: 4

# 93 "/root/.topspin-BladeEpu/local_acqu/ppDirs/zgesgp"

;use gradient ratio: gp 1 : gp 2

; 31 : 11

;for z-only gradients:

;gpz1: 31%

;gpz2: 11%

;use gradient files:

;gpnam1: SMSQ10.100

;gpnam2: SMSQ10.100

# 106 "/root/.topspin-BladeEpu/local_acqu/ppDirs/zgesgp"

;$Id: zgesgp,v 1.9 2012/01/31 17:49:32 ber Exp $
